# Development of Highly Fluorogenic Styrene Probes for Visualizing RNA in Live Cells

**DOI:** 10.1021/acschembio.3c00141

**Published:** 2023-05-18

**Authors:** Moon Jung Kim, Yida Li, Jason A. Junge, Nathan K. Kim, Scott E. Fraser, Chao Zhang

**Affiliations:** † Department of Chemistry & Loker Hydrocarbon Research Institute, University of Southern California, Los Angeles, California 90089, United States; ‡ Department of Biological Sciences, Division of Molecular and Computational Biology, University of Southern California, Los Angeles, California 90089, United States; § Translational Imaging Center, Michelson Center for Convergent Bioscience, University of Southern California, Los Angeles, California 90089, United States

## Abstract

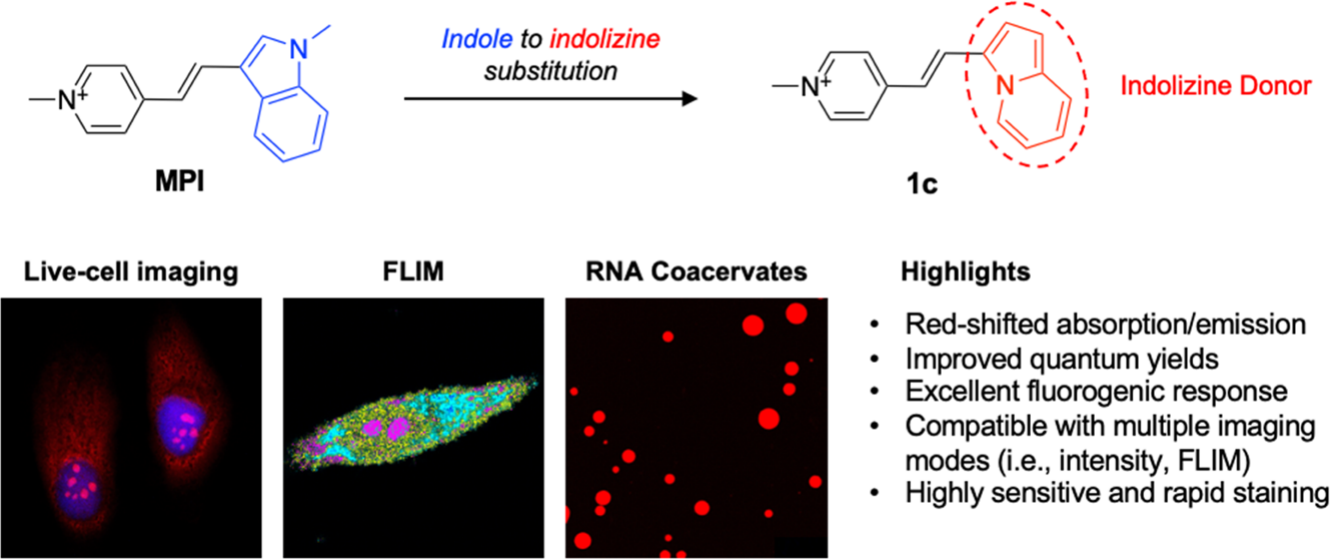

Styrene dyes are useful imaging probes and fluorescent sensors due to their strong fluorogenic responses to environmental changes or binding macromolecules. Previously, indole-containing styrene dyes have been reported to selectively bind RNA in the nucleolus and cytoplasm. However, the application of these indole-based dyes in cell imaging is limited by their moderate fluorescence enhancement and quantum yields, as well as relatively high background associated with these green-emitting dyes. In this work, we have investigated the positional and electronic effects of the electron donor by generating regioisomeric and isosteric analogues of the indole ring. Select probes exhibited large Stokes shifts, enhanced molar extinction coefficients, and bathochromic shifts in their absorption and fluorescence wavelengths. In particular, the indolizine analogues displayed high membrane permeability, strong fluorogenic responses upon binding RNA, compatibility with fluorescence lifetime imaging microscopy (FLIM), low cytotoxicity, and excellent photostability. These indolizine dyes not only give rise to rapid, sensitive, and intense staining of nucleoli in live cells but can also resolve subnucleolar structures enabling highly detailed studies of nucleolar morphology. Furthermore, our dyes can partition into RNA coacervates and resolve the formation of multiphase complex coacervate droplets. These indolizine-containing styrene probes offer the highest fluorescence enhancement among the RNA-selective dyes reported in the literature; thus, these new dyes are excellent alternatives to the commercially available RNA dye, SYTO RNASelect, for visualizing RNA in live cells and *in vitro*.

## Background

Selective labeling and visualization of cellular RNA has provided valuable knowledge and insight into RNA biology within the complex biological system.^1,2^ Over the years, significant efforts have been directed toward developing a variety of RNA labeling methods including, but not limited to, oligonucleotide-based hybridization probes, protein-based fluorescent reporters, RNA tagging with fluorogenic aptamers, and chemo-enzymatic modification of RNA.^3−8^ These developments have enabled a more precise investigation of RNA localization and dynamics. However, oligonucleotide- and protein-based methods often require expensive and complicated protocols as well as additional measures to allow for the successful delivery of the probes into cells (*i.e.*, microinjection, plasmid transfection, and/or Cu(I)-catalyzed azide–alkyne cycloaddition), thus greatly hampering their practical use for biological imaging.^9−12^

Small-molecule fluorescent dyes have emerged as powerful and versatile tools for studying biological systems.^13,14^ Low-molecular-weight dyes are easy to use and often display good cell permeability making them ideal for staining live cells without the need for fixation and permeabilization. Additionally, small molecules have superior chemical tractability and display tunable spectral and photophysical properties compared to oligonucleotide- and protein-based fluorescent probes.^13^ For these reasons, it is desirable to expand the current repertoire of fluorescent dyes for studying live-cell dynamics. There are many commercially available fluorescent dyes that populate the visible spectrum and stain a variety of organelles such as the nucleus (*via* binding chromosomal DNA), mitochondria, and lysosomes in live cells. However, cell-permeable small-molecule dyes for imaging RNA are severely lacking.^15^ To date, SYTO RNASelect (SYTO) is the only commercially available RNA dye compatible with live-cell imaging, yet its properties leave much to be desired.^16−18^ For instance, the excitation and emission wavelength of SYTO, which are in the blue and green regions of the visible spectrum (λ_ex_ = ∼490 nm; λ_em_ = ∼530 nm), are more susceptible to scattering and inducing phototoxicity in cells and exhibit significant background fluorescence in biological samples compared to longer-wavelength dyes. SYTO displays low photostability, poor aqueous solubility, and limited cell permeability, which restricts its use in time-sensitive and time-dependent imaging experiments. Finally, the structure of SYTO is undisclosed and its binding mode to RNA remains elusive, making it difficult to optimize its spectral and photophysical properties.

**Figure 1:**
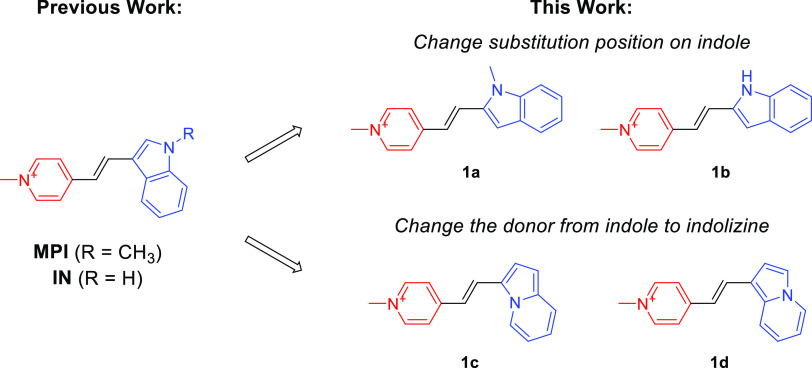
Chemical structures of the previously reported styrene-based RNA-selective dyes, MPI and IN, and the new dyes **1a**–**1d** reported in the current study. The electron donor and acceptor moieties are highlighted in blue and red, respectively.

Previous efforts from two research groups have been dedicated toward the development of two indole-based “push–pull” styrene fluorophores (MPI and IN), reported to selectively stain RNA in live cells.^19,20^ Methyl pyridinium indole (MPI) and IN both contain methyl pyridinium as the electron acceptor and an indole ring as the donor (Figure 1). In solution, free MPI and IN are virtually nonfluorescent, producing little background fluorescence. However, upon binding RNA, they exhibit greatly elevated fluorescence intensity. This attribute of “turn-on” fluorescence is particularly advantageous for sensing and imaging because fluorescence is only activated upon interaction with their biological targets, resulting in low background signals and high-contrast images.^21^ MPI and IN absorb and emit in the blue-green range of the visible spectrum (∼440 and ∼540 nm) with the former reported to have a fluorescence quantum yield of 32%, roughly double that of SYTO (17%). Although MPI was found to display more favorable photophysical properties (*e.g.*, higher photostability and larger Stokes shift) compared to SYTO, it was still difficult to generate high-contrast images because of its low quantum yield. Attracted by the small size and RNA selectivity of MPI-like dyes, we sought to improve the quantum yield, photostability, fluorogenic response, and emission wavelength of styrene dyes through chemical modifications.

We hypothesized that the spectral and photophysical properties of styrene dyes, such as MPI and IN, can be improved *via* modification of the indole donor. Herein, we tested this hypothesis by synthesizing and characterizing a panel of novel styrene dyes, which consist of regioisomeric and isosteric analogues of MPI and IN (Figure 1). The new styrene analogues not only retain their turn-on fluorescence upon binding RNA but also demonstrate red-shifted absorption and emission wavelengths. In particular, two indolizine-containing dyes displayed improved quantum yields, high photostability, and substantially greater fluorescence enhancement (>7-fold and >2-fold increase) upon binding RNA compared to MPI. These indolizine-containing dyes are also compatible with fluorescence lifetime imaging (FLIM) and can be used to resolve nucleoli in cells and multiphase separation in RNA coacervates.

## Results and Discussion

### Design and Synthesis of Dyes **1a**–**1d**

MPI and IN were used as the parental compounds in the design of new styrene dyes (Figure 1). We employed two strategies to modify the indole donor. One strategy involved retaining the indole donor and changing the substitution position on the indole ring while the other involved replacing the indole with its isostere, indolizine, another electron-rich arene. These strategies led to the design of two regioisomeric analogues (**1a** and **1b**) and two isosteric analogues (**1c** and **1d**) of MPI and IN.

**Scheme 1:**
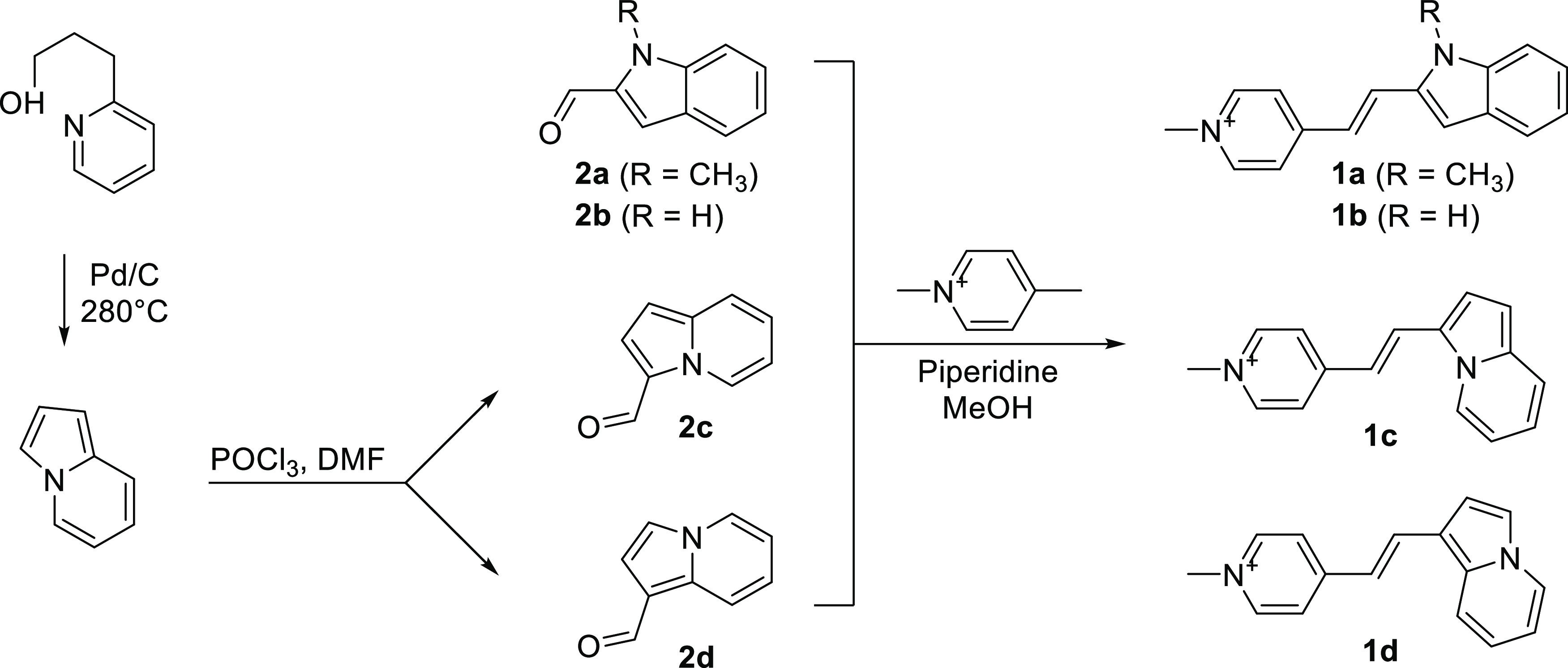
Synthesis of the Styrene Dyes **1a**–**1d**

The synthesis of compounds **1a**–**1d** is illustrated in Scheme 1. Indolizine was synthesized through a palladium-catalyzed intramolecular cyclization of 2-pyridinepropanol, and subsequently converted into the formyl indolizine intermediates (**2c** and **2d**) using the Vilsmeier-Haack reaction based on previously reported procedures.^22,23^ In the final step, the formyl-indoles (**2a** and **2b**) and formyl-indolizines (**2c** and **2d**) underwent a Knoevenagel condensation reaction with *N*-methyl-4-methyl pyridinium to yield the styrene probes **1a**–**1d**. The products were characterized by ^1^H NMR, ^13^C NMR, and MS (Supporting Information) with all of the spectroscopic data in agreement with the proposed chemical structures.

### Spectral and Photophysical Characterization of **1a**–**1d**

**Figure 2:**
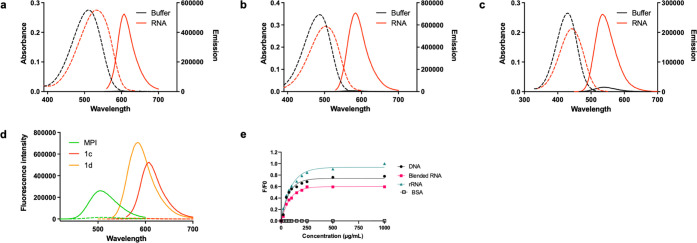
Absorption and emission spectra of (a) **1c**, (b) **1d**, and (c) MPI in T.E. buffer (pH 7.5) or RNA (Type IV RNA from torula yeast; 200 μg/mL). Dye concentration: 10 μM. Dashed and solid lines indicate absorbance and emission, respectively. (d) Comparison of fluorescence intensity between MPI, **1c**, and **1d** in buffer vs dyes in RNA solution (200 μg/mL) dye concentration 10 μM. Dashed lines reflect the fluorescence intensity of unbound dye, and solid lines indicate the fluorescence intensity of dyes in RNA solution. (e) Fluorescence titration of **1c** (10 μM) incubated with increasing concentration of nucleic acids or protein (BSA; bovine serum albumin).

After synthesis of the styrene dyes, their photophysical properties were evaluated. The spectral and photophysical properties (absorption, emission, molar absorptivity (ε), fluorescence quantum yield (Φ_F_), and fluorescence enhancement) of probes **1a**–**1d** were measured in DMSO, T.E. buffer (Tris–HCl, 1 mM EDTA, pH = 7.5), and RNA (200 μg/mL in T.E. buffer). The absorbance and emission spectra of **1a**–**1d** are shown in Figures 2a–c and S1a,b, and their photophysical properties are summarized in Table 1.

**Table 1:** Photophysical Properties of Styrene-Based Fluorescent Molecules

probe	solvent^a^	λ_abs_^b^	ε^c^	λ_em_^d^	Stokes shift (nm)	Φ_f_^e^^,^^f^(%)	fluorescence enhancement
**1a**	DMSO	436	29400	592	156	2.1	
	T.E.	422	20600	584	162	0.12	1
	RNA	434	19300	586	152	9.9	83
**1b**	DMSO	426	35400	570	160	1.3	
	T.E.	410	24400	568	142	0.12	1
	RNA	416	23300	552	136	3.9	33
**1c**	DMSO	518	36000	606	88	2.0	
	T.E.	510	27500	596	86	0.10	1
	RNA	556	27500	608	76	49	490
**1d**	DMSO	496	40300	586	90	3.3	
	T.E.	486	34600	572	86	0.38	1
	RNA	502	29400	584	82	50	132
MPI	DMSO	440	30500	538	98	3.7	
	T.E.	430	26500	540	110	0.52	1
	RNA	442	21200	534	92	32	62

a DMSO: dimethyl sulfoxide. T.E. buffer: 10 mM Tris–HCl, 1 mM EDTA, pH = 7.5. RNA: 200 μg/mL type IV torula yeast.

b Absorbance maximum wavelength (nm).

c Molar absorptivity (M^–1^ cm^–1^).

d Fluorescence maximum wavelength (nm).

e Fluorescence quantum yield of dyes using Coumarin 6 (Φ500 =0.78) and Rhodamine 6G (Φ548 = 0.95).

f Fluorescence quantum yield of dyes incubating with nucleotides using integrating sphere. Quantum yield data were averaged from duplicate experiments.

We observed slight solvatochromic shifts in the absorbance and emission spectra of **1a**–**1d** in DMSO and T.E buffer. These dyes are slightly more red-shifted in DMSO and RNA than in T.E. buffer. In T.E. buffer, **1a** and **1b** exhibited maximum absorbance wavelengths at 422 and 410 nm and a broad emission band centered at 584 and 568 nm, respectively, indicative of a charge transfer excited state.^24^ There are slight differences in the absorption and emission wavelengths of **1a** and **1b**, consistent with the minor structural and electronic differences between the two analogues. Dyes **1a** and **1b** exhibited large Stokes shifts of 162 and 142 nm with molar absorptivity values of 20,600 and 24,400 M^–1^ cm^–1^. As expected, the fluorescence quantum yields of the free dyes in solution were low (0.12% for **1a** and **1b**) which is consistent with the ability of styrene dyes to dissipate energy through rotation leading to nonradiative relaxation to the ground state.^25−27^ Direct comparison of the photophysical properties of regioisomers MPI and **1a** showed that both dyes shared similar absorption wavelengths while the emission wavelength of **1a** was significantly red-shifted (>50 nm) compared to that of MPI. The differences in the photophysical properties observed for the indole regioisomers may be explained by how electrons move through the conjugated π-system between the two resonance forms of the styrene dyes (Figure S10). Such qualitative analysis reveals that the electron transfer within **1a** and **1b** spans larger conjugated π-systems than that of MPI. Thus, the conjugated π-system of the 2-indole derivatives participates in electron transfer to a higher degree than those of the 3-indole derivates. This may account for the observed red-shifted emission of **1a** and **1b** compared to MPI.

The indolizine dyes **1c** and **1d** displayed a significant red shift in both absorption (510 and 486 nm) and emission (596 and 572 nm) wavelengths (Figure 2a,b and Table 1). The size difference between the conjugated π-system undergoing electron movement may account for the observed bathochromic shift of the indolizine dyes. Dyes **1c** and **1d** show narrow emission bands and moderate solvatochromic effects which supports that emission occurs from a locally excited state.^28,29^**1c** and **1d** displayed a large Stokes shift of 86 nm and molar absorptivity values of 27,500 and 34,600 M^–1^ cm^–1^. As expected, the fluorescence quantum yields of the free dyes in T.E. buffer were low, with values at 0.10 and 0.38% for **1c** and **1d**, respectively.

### Fluorogenic Response of **1a**–**1d** to RNA *In Vitro*

To determine the fluorescence change of **1a-1d** in the presence of RNA, we incubated the dyes (10 μM) with torula yeast type IV RNA in T.E. buffer and measured the spectral and photophysical parameters as described above. Fold-enhancement values were calculated by taking the ratio of the quantum yield of dyes in RNA solution over free dyes. Dyes **1a** and **1b** exhibited an 83- and 33-fold fluorescence enhancement with modest quantum yields of 9.9 and 3.9%, respectively (Table 1 and Figure S1a,b). While **1a** displayed a similar degree of fluorescence enhancement compared to MPI, **1b** displayed only a 33-fold fluorescence enhancement, roughly half the observed fluorescence enhancement of MPI. This data suggests that a methyl substituent on the indole nitrogen can enhance quantum yields possibly due to the electron-donating effect imparted by the methyl group.

The indolizine dyes **1c** and **1d** showed a significant increase in fluorescence intensity upon addition of RNA with absolute quantum yield values of 49 and 50% (Table 1 and Figure 2a,b). We observed a remarkable 490- and 132-fold fluorescence enhancement of **1c** and **1d** in response to binding RNA. Notably, the quantum yield and fluorogenic response of indolizine-containing dyes (**1c** and **1d**) are much higher than those of indole-containing dyes (**1a**, **1b**, and MPI). When **1c** was incubated with DNA, it generated a lower fluorogenic response than dyes incubated with RNA (Figure 2e), indicating a moderate selectivity for RNA over DNA *in vitro*. Upon incubation with the same RNA species, **1c** demonstrated a substantially higher fluorogenic response than SYTO RNASelect (Figure S1c). Additionally, we confirmed that **1c** can stain RNA in PAGE-gels, although its sensitivity was lower than the commercial standard, SYBR Gold (Figure S2). We speculate that the smaller size of **1c** than commercial cyanine dyes, such as SYBR Gold, may be responsible for its comparatively lower sensitivity *in vitro* when staining RNA in gels. Given that **1c** and **1d** display the most favorable spectral and photophysical qualities among our styrene dyes, we chose to further characterize these indolizine dyes in subsequent *in vitro* and cell imaging experiments.

### Live- and Fixed-Cell Imaging Using **1c** and **1d**

**Figure 3:**
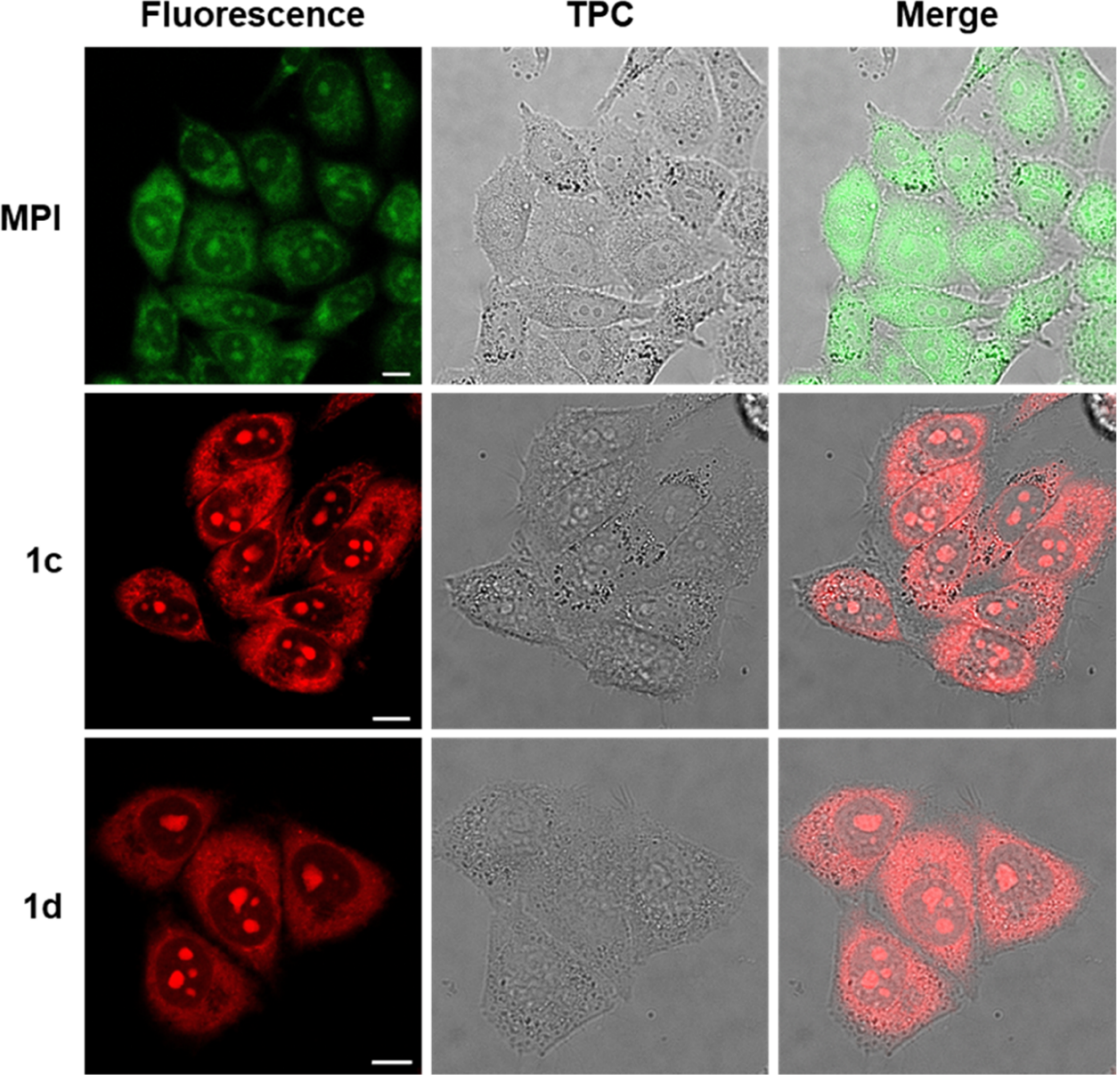
Confocal fluorescence images of live HeLa cells incubated with 20 μM MPI (λ_ex_ = 470 nm, λ_em_ = 525–555 nm), **1c** (λ_ex_ = 550 nm, λ_em_ = 580–620 nm), and **1d** (λ_ex_ = 500 nm, λ_em_ = 560–600 nm) for 30 min. TPC: transmission phase contrast. Scale bar: 10 μm.

To determine the cellular localization of **1c** and **1d**, live HeLa cells were stained with 20 μM of either dye for 30 min prior to image acquisition. Fluorescence signals of **1c** and **1d** can be seen primarily within the nucleoli and cytoplasm (Figure 3), and the nucleus is otherwise dark, consistent with preferential binding of the dyes to RNA. The localization of **1c** and **1d** was similar to HeLa cells stained with MPI. The intensity and contrast of HeLa cell images obtained using **1c** or **1d** are substantially higher than those of MPI, consistent with the higher quantum yield and fold-enhancement observed with the indolizine dyes (Table 1). Viewing the fluorescence images of **1c** at higher magnification, we observed distinguishable fiber-like structures distributed throughout the cytoplasm that resemble mitochondrial staining profiles (Figure S3). This can be explained by the lipophilic, cationic character of **1c**, which allow for accumulation in the mitochondria similar to established mitochondrial dyes (*e.g.*, MitoView and MitoTracker) due to the negative mitochondrial membrane potential.^30,31^ The validity of these structures as mitochondria was further confirmed through our dynamic imaging data, which exhibited their movement within the cytoplasm (Supporting Video 1).

Our dyes displayed excellent kinetics and sensitivity for imaging nucleoli in live cells. Nucleoli could be clearly discerned as early as 30 s after addition of either **1c** or **1d** in the medium, while no signals from SYTO could be detected even after 30 min of incubation following the manufacturer’s protocol (Figure S4). A dose–response study revealed that nanomolar concentrations of **1c** were sufficient for visualizing nucleoli in live HeLa cells (Figure S5). Incubating cells with higher concentrations of SYTO resulted in dye aggregates that can be seen as bright droplets in the medium (Figure S5b).

To determine whether dyes **1c** and **1d** show similar localization profiles under fixed conditions, HeLa cells were fixed with 4% paraformaldehyde (PFA) before staining with either dye. We found that **1c** and **1d** show qualitatively similar labeling patterns as observed with live-cell imaging, with fluorescence signals predominantly coming from the nucleoli and cytoplasm. As expected, fixation abolished the mitochondrial membrane potential and the fiber-like appearance of the staining was absent in the cytoplasm. The nucleolar staining of **1c** was confirmed to be RNA-dependent *via* an experiment in which fixed HeLa cells were treated with RNase (Figure S6). Additionally, co-staining HeLa cells with SYTO and **1c** resulted in co-localization of the two dyes in nucleoli (Figure S7).

**Figure 4:**
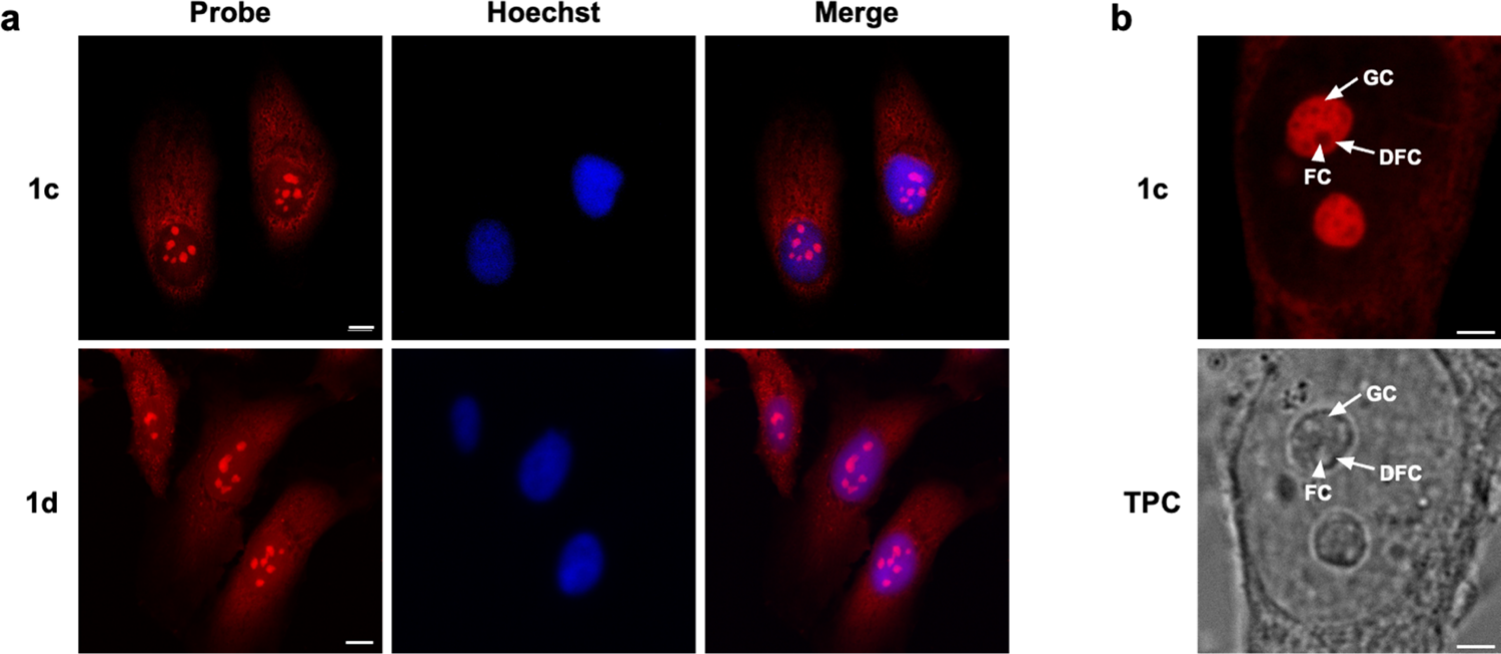
(a) Confocal fluorescence images of PFA-fixed HeLa cells incubated with 20 μM **1c** and **1d** for 30 min and 1 μg/mL Hoechst 33342 for 30 min. Scale bar: 10 μm. (b) Zoomed-in image of HeLa cells stained with 20 μM **1c** for 30 min. Image shows the subnucleolar components FC (indicated by the white arrowhead), DFC, and GC (indicated by the white arrows). TPC: transmission phase contrast. Scale bar: 3 μm.

To assess the counterstaining compatibility of probes **1c** and **1d**, we stained HeLa cells with either **1c** or **1d** and Hoechst 33342 (Figure 4a). Fluorescence signals from the dyes can be clearly seen within nucleolar and cytoplasmic structures while Hoechst 33342 signals are restricted within the nucleus, as expected. The data suggest that fluorescence signals from **1c**-**1d** and Hoechst 33342 are distinct and that the two types of dyes are compatible in co-staining experiments.

We investigated whether our indolizine dyes could resolve the substructures of the nucleolus. Morphologically, the nucleolus is divided into a fibrillar center (FC), a dense fibrillar component (DFC), and an outer granular component (GC) all with distinct functions in ribosome biogenesis.^32^ Enlarged images of the nucleolus clearly show **1c** is able to reveal distinct subnucleolar regions by fluorescence microscopy (Figure 4b). The images show the GC intensely stained by **1c** while the FC and DFC appear as much dimmer cavities within the nucleolus. These observations can be attributed to the presence of RNA within the subnucleolar structures. The GC, rich in rRNA and ribosomal proteins, contain pre-ribosomal subunits while the FC contains clusters of condensed rDNA chromatin.^33,34^ The fluorescence images obtained using **1c** are corroborated by transmission phase contrast, in which the nucleolar structures can be discerned albeit with lower resolution (Figure 4b).^32,35^ These data suggest that **1c** can be used to visualize and study subnucleolar structures and nucleolar morphology in live and fixed cells.

**Figure 5:**
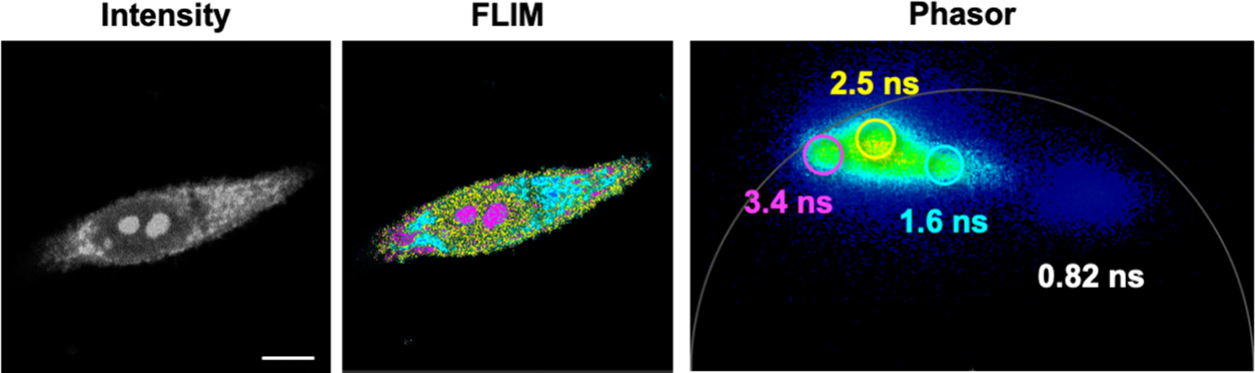
Comparison of intensity and FLIM phasor processed images of HeLa cells stained with **1c** (20 μM). Separation of lifetimes was performed using FLIM phasor analysis, in which very short lifetimes appear on the bottom right and very long lifetimes appear on the bottom left. The phasor plot shows four unique lifetimes (color-coded on the FLIM image), corresponding to fluorophores within the nucleolus, nucleus, cytoplasm, and surrounding media. Scale bar: 10 μm.

To further characterize our dyes in cells, we performed fluorescence lifetime imaging microscopy (FLIM) to study whether our dyes display unique fluorescence lifetimes in cells. We observed four distinct fluorescence lifetime species of **1c** depending on its subcellular locations: nucleolus (3.4 ns), nucleus (2.5 ns), cytoplasm (1.6 ns), and unbound dye (0.82 ns). We utilized the phasor approach to FLIM analysis, as it provides a powerful fit-free tool to characterize and display differences in dye microenvironment through a graphical interface with imaging data.^36,37^Figure 5 shows a side-by-side comparison of intensity-based and FLIM phasor masked images, highlighting differences in subcellular RNA-positive microenvironments. Dyes like **1c** with unique and separable lifetimes are valuable tools for differentiating multiple RNA-containing structures that are stained by the same dyes and for monitoring the dynamics of the RNA-containing structures at those locations. These data suggest that FLIM can provide a second dimension to distinguish the different cellular structures stained by the same indolizine dye.

### Cytotoxicity and Photostability

**Figure 6:**
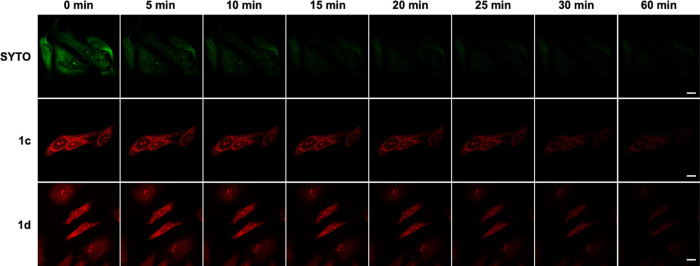
Comparison of photobleaching of SYTO RNASelect, **1c**, and **1d**. Confocal fluorescence images of PFA-fixed HeLa cells incubated with **1c** and **1d** (1 μM) and SYTO RNASelect (1 μM). Scale bar: 20 μm.

We evaluated the cytotoxicity of **1c** and **1d** by performing an MTT assay using HeLa cells. The cells were incubated with each dye at concentrations ranging from 0.1 to 30 μM for 24 h. Our results show that >70% of the cells remained viable after 24 h of incubation with 30 μM of either **1c** or **1d** (Figure S8). These indolizine-containing probes can thus be considered largely nontoxic for short-term imaging experiments. It is important to note that cytotoxicity results from repeated or prolonged exposure of fluorescently labeled cells to irradiation from high laser powers.^38^ Cells overexposed to irradiation may sustain damage to macromolecules and organelles which can negatively influence cell viability. Several studies have reported that red-shifted dyes are preferable to shorter-wavelength dyes (*e.g.*, violet or blue excitation) due to the lower incidence of cell death.^39−41^ To minimize toxicity, several factors must be considered: the dye concentration, laser power, and excitation wavelength. In our investigation, we determined that **1c** could be used at concentrations as low as 10 nM to resolve nucleoli while SYTO RNASelect required higher concentrations (>2 μM) to see fluorescent signals (Figure S5). Furthermore, the favorable photophysical properties of **1c** (*e.g.*, high quantum yield) allow for the facile acquisition of high-contrast live-cell images with low laser power, while the same could not be achieved with SYTO. Finally, the excitation wavelength of both **1c** and **1d** are relatively more red-shifted compared to SYTO (Table 1). Having longer excitation wavelengths comes with many advantages including reduced photobleaching (Figure 6), increased tissue penetration, and reduced autofluorescence.^42^

We evaluated the photostability of our dyes compared to SYTO. Fixed HeLa cells were incubated with 1 μM **1c**, **1d**, and SYTO for 30 min and thoroughly washed to remove excess dye. The cells were exposed to continuous irradiation under a fixed laser power and imaged over a range of different time points (Figure 6). We quantitatively analyzed the fluorescence intensities at each time point and determined that **1c** and **1d** displayed significantly higher photostability, with a half-life (t_1/2_) of ∼13 min. In contrast, SYTO displayed a t_1/2_ of ∼1.5 min which is >8-fold less than that of **1c** and **1d** (Figures 6 and S9). These results suggest that **1c** and **1d** are suitable for longer imaging experiments without the issue of photobleaching.

### Detection of RNA Coacervates Using **1c**

Liquid-liquid phase separation (LLPS) has emerged as a new paradigm in the study of cellular processes.^43^ LLPS is thought to be the underlying mechanism behind the formation of intracellular membraneless organelles (MLO) such as nucleoli and P granules.^43−45^ Studying these MLOs can provide insight into the molecular basis of disease.^44,46^ Thus, research efforts have focused on understanding their formation for further investigation of the physiology and pathophysiology of a wide range of biological processes and systems.^45^ The most common method used to initially detect LLPS is microscopy. Hence, the use of fluorescently labeled condensate components can enable their detection *in vitro* and in cells. Previous studies have utilized fluorescently labeled RNA,^47,48^ peptides,^49^ and proteins^50−52^ to visualize RNA coacervates, a type of droplet formed by LLPS. However, these strategies require modifying RNA and engineering proteins with exogenous fluorophores such as fluorescein and GFP. Given that **1c** exhibits a remarkable fluorogenic response upon binding RNA, we hypothesized that **1c** could enrich and label RNA coacervates. To test this, we devised a simple *in vitro* model consisting of torula yeast RNA (negative polyelectrolyte) and spermine (positive polyelectrolyte) following an adapted procedure.^47,48^ The coacervates were incubated with **1c** and imaged using confocal fluorescence microscopy and FLIM.

**Figure 7:**
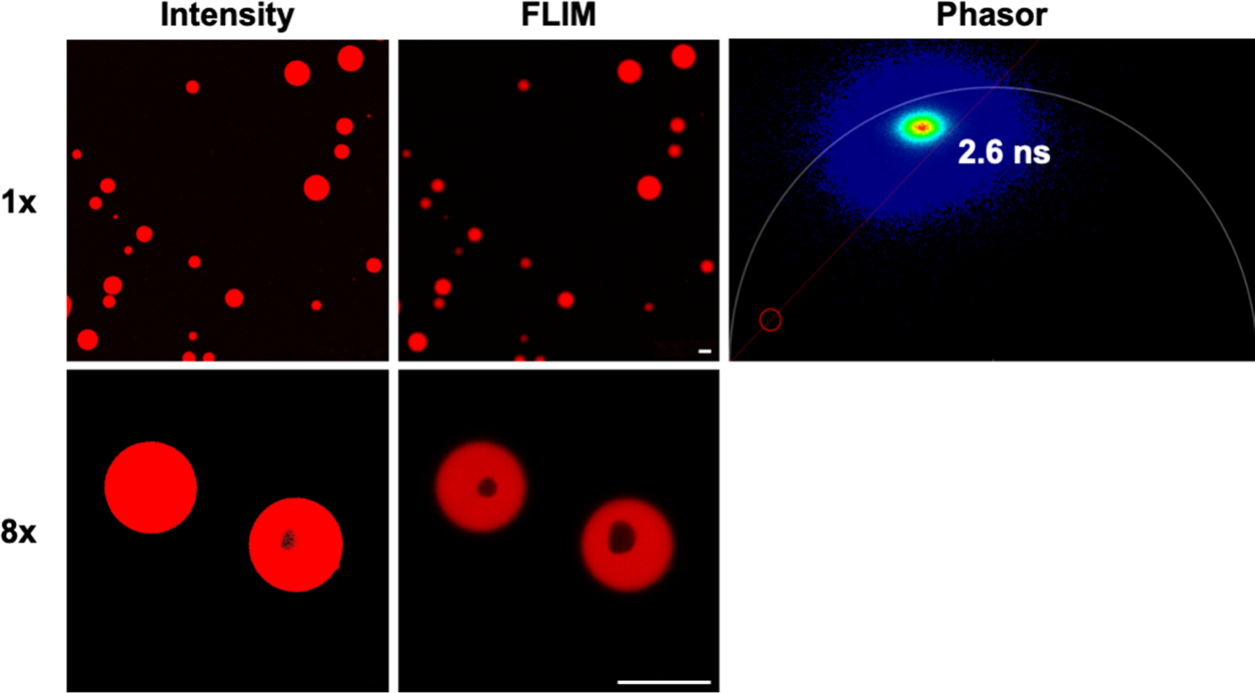
Partitioning of **1c** (10 μM) within the RNA/spermine condensates. The top row shows images enlarged by 1×, while the bottom row shows images enlarged by a factor of 8×. The left column shows images based on fluorescence intensity alone, and the middle column shows images resolved using FLIM. The phasor plot indicates the presence of a single lifetime species of **1c** (2.6 ns). Scale bar: 10 μm.

We observed the formation of spherical coacervate droplets upon mixing the RNA and spermine solutions in a high ionic strength buffer. Coacervates incubated with **1c** were visualized using confocal fluorescence microscopy and FLIM (Figure 7). Both intensity and FLIM images show that **1c** readily partitions into RNA coacervates and exhibits intense fluorescence signals where RNA is densely concentrated. We attribute this to the cationic and lipophilic nature of **1c** which favors accumulation in hydrophobic and water-poor regions while the strong fluorogenic response is due to reduced rotational freedom of the probe when bound to RNA. The phasor plot indicates the presence of a single fluorescence lifetime species which is evenly dispersed throughout the coacervate. In addition to the uniform coacervate droplets, multiphase complex coacervates are also formed under these conditions due to sufficient differences in macromolecular density driven by charge-charge interactions and critical salt concentrations.^51^ Interestingly, FLIM can better resolve the multiphase complex RNA coacervates (cavity-containing droplets) than that of intensity-based imaging. The different layers in the co-existing phases present distinct chemical environments that can concentrate **1c** or other guest molecules to different extents. We speculate the cavity observed in Figure 7 to be a highly solvated region where fluorescence signals are quenched due to nonradiative decay. Further experiments would be needed to fully characterize the chemical environment of each phase.

In conclusion, we developed a panel of fluorogenic styrene dyes for visualizing RNA in live cells and in RNA coacervates. Given that MPI has previously shown good cell permeability and RNA selectivity, but displays only moderate fluorescence enhancement and quantum yield, we sought to generate analogues of MPI to improve its spectral and photophysical properties while retaining its selectivity for RNA in cells. By changing the substitution position on the indole donor and replacing the indole with indolizine, we generated four novel styrene dyes **1a**–**1d** which exhibited significantly altered spectral and photophysical profiles. Dyes **1a** and **1b** are mere regioisomers of MPI yet they displayed >20 nm red shift in emission wavelengths and significantly larger Stokes shifts than MPI. The positional change of the indole donor from the 3- to 2- position present an altered electronic configuration that involves a greater area in the conjugated π-system of the dye scaffold and thus lowers the overall energy of the molecule (Figure S10). Apart from the significant red-shifted emission wavelengths, dyes **1a** and **1b** displayed, on average, lower molar absorptivity and quantum yield values than that of MPI. Despite the excellent 83-fold fluorescence enhancement observed for **1a**, its utility is limited due to its low quantum yield.

In contrast, when the indole donor was replaced with indolizine, the resulting indolizine-containing dyes exhibited improvements in not only the spectral properties but also the photophysical properties. These dyes were found to absorb and emit in the red region of the visible spectrum, making them more ideal for imaging cells and tissues. Moreover, **1c** and **1d** displayed high quantum yields and a remarkable fluorescence enhancement upon binding RNA. We reasoned that the donor replacement resulted in an expansion of conjugation in electron transfer to lower the overall energy of the system—similarly to what was observed for **1a** and **1b** (Figure S10). Rigorous computational studies are currently underway to explain the superior properties of indolizine over indole among these styrene dyes and will be reported soon.

Indolizine is a nitrogen-containing heterocycle that has found many uses in medicinal chemistry and pharmaceuticals.^53,54^ Recently, indolizines have been explored for applications in fluorescent and luminescent materials, notably in organic light-emitting diodes (OLEDs) because of their high quantum yield and tunable fluorescence properties.^55−57^ Our study incorporates the indolizine moiety into a styrene scaffold for labeling RNA in live cells. Our data validate the excellent photophysical properties of indolizines especially compared to its indole isostere. To our knowledge, this study represents the first application of this interesting heterocycle in RNA-selective dyes for live-cell labeling.

We have demonstrated that **1c** and **1d** are compatible with both live- and fixed-cell imaging experiments and can resolve subnucleolar structures such as the FC from the surrounding GC. Additionally, our dyes have been used to capture dynamic cellular processes including mitochondria trafficking and apoptosis (Supporting Videos 1 and 2). Co-staining experiments with Hoechst 33342 suggests that our dyes are compatible with nuclear stains and are likely compatible with other organelle-specific dyes. Our data shows that **1c** and **1d** indeed label RNA-rich nucleoli with high sensitivity, rapid labeling kinetics (Figure S4), high contrast, and low background. We observed co-localized signals of SYTO and **1c** in nucleoli indicating both dyes are associated with RNA-rich nucleoli. However, we do not observe fluorescence signals in the surrounding nucleus suggesting that **1c** and **1d** do not bind to chromosomal DNA *via* intercalation or minor groove binding. Although the exact mechanism for the binding of these styrene dyes to RNA has not been elucidated; based on our observations we hypothesize that **1c** and **1d** may *not* act as classic intercalators or minor groove binders upon binding RNA. To further improve upon these dyes, efforts must be placed in elucidating the exact binding mode of these dyes to RNA.

In addition to cell-based imaging, we show that our dyes can selectively partition and label RNA coacervates *in vitro*. Developing small-molecule probes that can selectively accumulate in coacervates is instrumental in studying LLPS and can serve as key tools in delineating the function of biomolecular condensates in cells and their physiological and pathophysiological roles.

Our dyes display good photostability and are noncytotoxic, making them useful for long-term, time-resolved imaging experiments. The excellent quantum yields of **1c** and **1d** negate the use of high laser powers to irradiate the dyes allowing for reduced incidence of phototoxicity. Given the high sensitivity of our dyes, concentrations as low as 10 nM were successfully used to resolve nucleoli (Figure S5). Our dyes display unique fluorescence lifetimes within distinct cellular and aqueous environments (*i.e.*, nucleolus, nucleus, and cytoplasm) allowing for selective imaging based on fluorescence lifetimes. We envision our dyes to serve as excellent probes for FLIM experiments to resolve cellular structures beyond fluorescence intensity and emission wavelength. In fact, our dyes may even be compatible with other red-colored dyes (λ_em_ > 580 nm) regardless of spectral overlap. Given the excellent fluorescence properties of the novel indolizine dyes, we believe that they can serve as a better alternative to SYTO RNASelect in a wide variety of cell imaging studies. Finally, we aim to expand the utility and application of these RNA-selective fluorogenic dyes. Current efforts have been directed toward extending our library of styrene dyes by incorporating additional electron donors and acceptors onto the styrene scaffold. Furthermore, we aim to incorporate chemical moieties that can enable selective binding to RNA species of specific sequences.

## Materials and Methods

### Chemical Synthesis

See the Supporting Information for a detailed description of experimental methods.

### Quantum Yield Measurements of **1a**–**1d** in T.E. Buffer and RNA Solution

Coumarin 6 was used as a reference dye for MPI, **1a**, and **1b** and Rhodamine 6G was used as a reference for **1c** and **1d**. Absorption spectra were collected by Shimadzu UV-1800 spectrophotometer. Samples were loaded in plastic disposable cuvette. Emission spectra were obtained by Photon Technology International QuantaMaster model C-60 Fluorimeter in 1 × 1 cm^2^ quartz cuvettes. Fluorescence quantum yields were then calculated according to the method by Lawson-wood, Upstone, and Evans.^58^ The torula yeast type IV RNA (Sigma) solution was prepared as a 200 μg/mL solution in T.E. buffer (Tris–HCl, EDTA, pH 7.6, Bioworld) without sonicating to avoid shearing of nucleic acids. All measurements were taken using samples with a final dye concentration of 10 μM.

### Fluorescence Titration of **1c**

Solutions of torula yeast RNA type IV (RNA mixture) and DNA from calf thymus (Sigma) were prepared and stored at 4 °C overnight. Bovine serum albumin (BSA, Thermo Fisher) and Baker’s yeast RNA (rRNA, Sigma) solutions were prepared at 2 h before testing. 100 μL of 1 μM **1c** were diluted by corresponding substrates solution reaching concentrations ranging from 0 to 1000 μg/mL of nucleic acids or BSA. The resulting mixtures were placed in a 96-well opaque plate. The plate with the mixture was gently shaken for 5 min before being tested by a microplate reader.

### Comparison with SYTO RNASelect

100 μL of 1 μM **1c** and SYTO RNASelect were diluted by 100 μL solutions of RNA mixture to reach final concentrations ranging from 0 to 1000 μg/mL. The resulting mixtures were placed in a 96-well opaque plate. The plate with the mixture was gently shaken for 5 min before being tested by a microplate reader (SpectraMax iD5).

### General Method for Cell Culture

HeLa cells were cultured in Dulbecco’s modified Eagle’s medium (DMEM, Thermo Fisher) supplemented with 10% (v/v) fetal bovine serum (FBS, Thermo Fisher) and incubated at 37 °C with 5% CO_2_.

### Live-Cell Imaging

HeLa cells were cultured at a density of 7000 cells/well on chambered glass sides and incubated at 37 °C overnight or until fully adherent. After removing the medium and washing with DPBS (Thermo Fisher), the cells were incubated with dyes in PBS (Thermo Fisher) for 30 min at 37 °C with 5% CO_2_ on the microscope stage and analyzed directly. Fluorescence images were acquired using a confocal microscope (Leica SP8, Leica Microsystems) and analyzed using ImageJ. MPI (20 μM) was excited at 440 nm, SYTO RNASelect (0.1–1 μM, Thermo Fisher) was excited at 490 nm, **1c** (10 nM–20 μM) was excited at 550 nm, and **1d** (20 μM) was excited at 500 nm.

### Time-Resolved Live-Cell Imaging

HeLa cells were cultured on eight-well chambered glass slides (iBidi μ-slides) and incubated overnight at 37 °C with 5% CO_2_ until totally adherent. Staining solutions for each dye were prepared in serum-free DMEM and kept warm at 37 °C. The cells were monitored from 0 to 30 min, where *t* = 0 is the time right before the addition of the dye. Images were acquired at *t* = 0, 0.5, 1, 2, 5, 10, 15, 20, 25, and 30 min for each dye. Fluorescence images were analyzed and quantified with ImageJ.

### Fixed-Cell Imaging

HeLa cells were cultured on 6-well plates containing glass coverslips and incubated at 37 °C overnight or until fully adherent. After removing the medium and washing with DPBS, the cells were fixed with 4% paraformaldehyde for 10 min at ambient temperature. After fixation, the cells were rinsed with PBS and incubated with dye solutions in PBS for 30 min at ambient temperature. After incubation, the dye solutions were removed and cells were gently washed with PBS three times. The coverslips were mounted onto glass slides, sealed, and analyzed using confocal microscopy.

### Ribonuclease (RNase) Digest Experiment

HeLa cells were cultured in six-well plates with glass coverslips and incubated at 37 °C overnight to totally adherent. The cells were fixed according to the protocol described above with minor modifications. A solution of 4% paraformaldehyde + 0.1% Triton-X was prepared in PBS and used to fix and permeabilize HeLa cells. The cells were incubated with this solution for 20 min at room temperature. After rinsing the cells with PBS, the cells were stained with 1 μM **1c**, **1d**, or SYTO in PBS for 30 min at room temperature. After removing the staining solution, 0.5 mL of RNase (100 μg/mL) and PBS (negative control) was added into each respective well and incubated for 6 h. After incubation, the medium was removed, and the cells were thoroughly washed with PBS three times. The glass coverslips were mounted onto glass slides, sealed, and analyzed using confocal microscopy.

### Counterstaining with Hoechst 33342

HeLa cells were cultured in six-well plates with glass coverslips and incubated at 37 °C overnight to totally adherent. The cells were fixed according to the protocol described above and stained with 20 μM **1c** or **1d** and 1 μg/mL Hoechst 33342 (Thermo Fisher) diluted in PBS for 30 min in the dark. After incubation with dyes, the cells were gently washed with PBS. The coverslips were mounted onto glass slides, sealed, and analyzed using confocal microscopy.

### Photostability

HeLa cells were fixed, stained with 1 μM **1c** and SYTO RNASelect, and mounted according to the protocol described above. The cells were analyzed using confocal microscopy and continuously irradiated at 550 nm (**1c**) and 490 nm (SYTO RNAselect) holding a fixed laser power. Images were taken at indicated time points (0, 5, 10, 15, 20, 25, 30, and 60 min). Fluorescence intensities were quantified using ImageJ.

### *In Vitro* Assay for Cytotoxicity

HeLa cells were cultured (2000 cells/well) in DMEM and supplemented with 10% FBS in 96-well plates. The solution of MPI, **1c**, and **1d** at indicated concentrations (0, 0.1, 0.3 1, 3, 10, 30 μM) were added to each well diluted with DMEM and cells were incubated for 24 h at 37 °C with 5% CO_2_. The next day, 10 μL of MTT (Abcam) labeling reagent was added to the pretreated cells and incubated for 4 h. A control was prepared in the same manner by adding 10 μL of MTT labeling reagent to untreated HeLa cells. After incubation, 100 μL of solubilization reagent was added to each well and the plate was shaken at 37 °C for 15 min in an orbital shaker. Upon complete solubilization of the purpose formazan crystals, the absorbance of the samples was measured at 570 nm using a microplate reader. The absorbance of each sample was normalized with its control. MTT assays were performed in triplicates.

### RNA-Spermine Condensate Preparation

A 1 wt % stock solution of torula yeast RNA type IV was prepared in nuclease-free water and stored in multiple aliquots at −20 °C. Final concentrations of RNA ranged from 0.02 to 0.4 wt %. A 1 wt % stock solution of spermine was prepared in deionized water and stored at 4 °C. The spermine (Sigma) concentration was fixed at 0.1 wt %. Dye **1c** was prepared as 10 mM stock solutions in DMSO. Condensates were prepared in a 5 mM HEPES (pH 7.4, VWR), 1 mM MgCl_2_ buffer. Stock solutions were added in the following order for the preparation of each sample: deionized water, HEPES, MgCl_2_, RNA, and spermine. Samples were mixed *via* gentle pipetting in between the addition of each component.

### Staining and Imaging of Condensates

RNA condensates were incubated with 10 μM **1c** for 1 min at room temperature. To image the condensates, 20 μL of the incubated sample was added onto a glass coverslip and mounted onto a glass slide. Images were acquired using fluorescence confocal microscopy and FLIM.

### Native-PAGE Gel Staining

1 L of 10× TBE running buffer was prepared by mixing 108 g of Tris Base, 55 g of boric acid, and 40 mL of 0.5 M EDTA (Apex). The combined solution was diluted to 1 L by filtered H_2_O. 14 mL of 8% Native-PAGE gel was prepared by mixing 0.7 mL of 10× TBE buffer, 0.14 mL of 10% APS buffer (Bio-Rad), and 3.73 mL of 30% acrylamide solution. The mixture was diluted to 14 mL with filtered water. 28 μL of TEMED (Sigma) was added to the mixture. The gel mixture was transferred into the casting frame placed in a precooled gel chamber. An 18-well cast was put on the top and left for polymerization for 15 min on ice. The gel chamber was filled with cold 0.5× TBE running buffer. The gel was pre-run at 150 V for 40 min. The RNA marker mixture was loaded (5 μL micro-RNA marker + 0.4 μL low range marker (Bio-Rad) for one well). 5 μL of rRNA sample was loaded to reach rRNA amount as 1 and 10 μg. The RNA marker and rRNA sample loading had four repeats on the same gel. The gel was run at 150 V for 30 min. The cut gels were stained and shaken in 25 mL of 1× SYBR Gold (Thermo Fisher), 2 μM **1c**, 20 μM **1c**, and 40 μM **1c** for 15 min. The gels were de-stained with 0.5× TBE buffer and shaken for 10 min. The buffer was removed, and the gels were imaged using an Amersham Typhoon Biomolecular Imager.

## Supporting Information Available

Photophysical spectra; gel staining; details of chemical synthesis; NMR spectra; MS spectra; time-resolved fluorescence images; fluorescence images of MitoTracker; fluorescence images with RNase treatment; fluorescence images of live Hela cells incubated with various concentrations of **1c** and SYTO RNASelect; and cytotoxicity and graphical presentation of conjugated structures of our dyes (PDF)
Dynamic imaging of live HeLa cells (MP4)
Dynamic imaging of live HeLa cells undergoing apoptosis (MP4)
