# Supplementary material for: Development of Highly Fluorogenic Styrene Probes for Visualizing RNA in Live Cells
Source: ACS Chem Biol. 2023 May 18;18(7):1523–33. doi: 10.1021/acschembio.3c00141 (PMC10367048; doi:10.1021/acschembio.3c00141)
Supplement: Supplementary file 1 — cb3c00141_si_001.pdf [file cb3c00141_si_001.pdf]

Supporting Information

Development of highly fluorogenic styrene probes for visualizing RNA in live cells

Moon Jung Kim[1§], Yida Li[1§], Jason A. Junge[2,3], Nathan K. Kim[1], Scott E. Fraser[2,3], Chao Zhang[1,2*]

[1]Department of Chemistry and Loker Hydrocarbon Research Institute, University of Southern California, Los Angeles, CA 90089, United States

[2]Department of Biological Sciences, Division of Molecular and Computational Biology, University of Southern California, Los Angeles, CA 90089, United States

[3]Translational Imaging Center, Michaelson Center for Convergent Bioscience, University of Southern California, Los Angeles, CA, 90089, United States, United States

[§]Co-first authors with equal contributions

[*]Correspondence to zhang.chao@usc.edu

Supplemental Figures

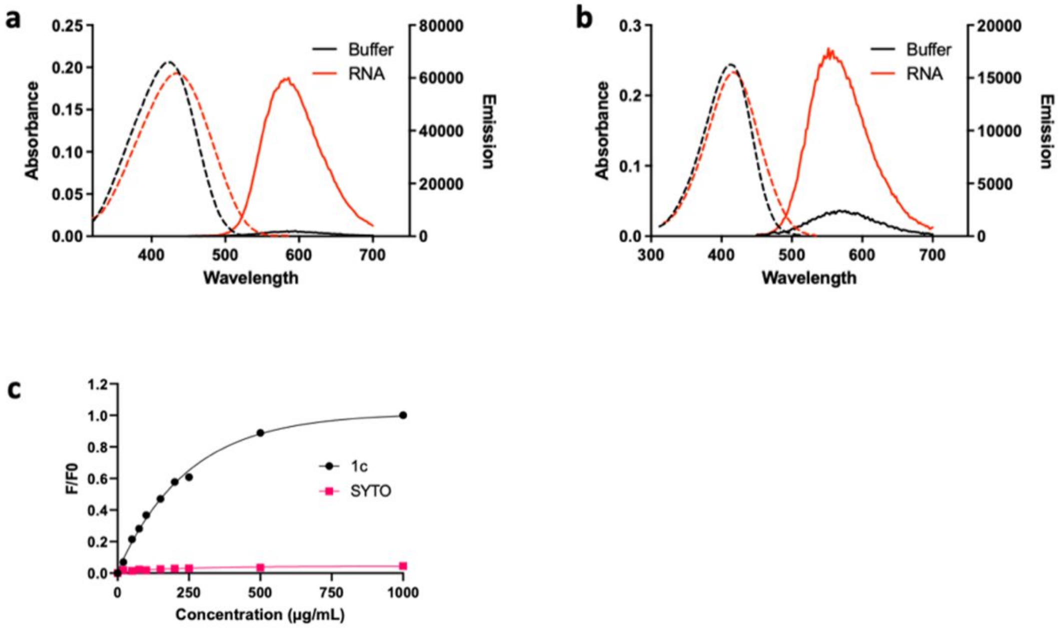

Figure S1. Absorption and emission spectra of the styrene dyes (a) **1a** and (b) **1b** in T.E. buffer (pH 7.5) or RNA (type IV RNA from torula yeast; 200 μg/mL). Dye concentration: 10 μM. (c) Fluorescence titration of **1c** and SYTO^{TM} RNASelect with increasing concentration of rRNA. Baker's yeast RNA that is rich in ribosomal RNA was used as the "rRNA" The dyes **1c** and SYTO were used at 1 μM in the experiments.

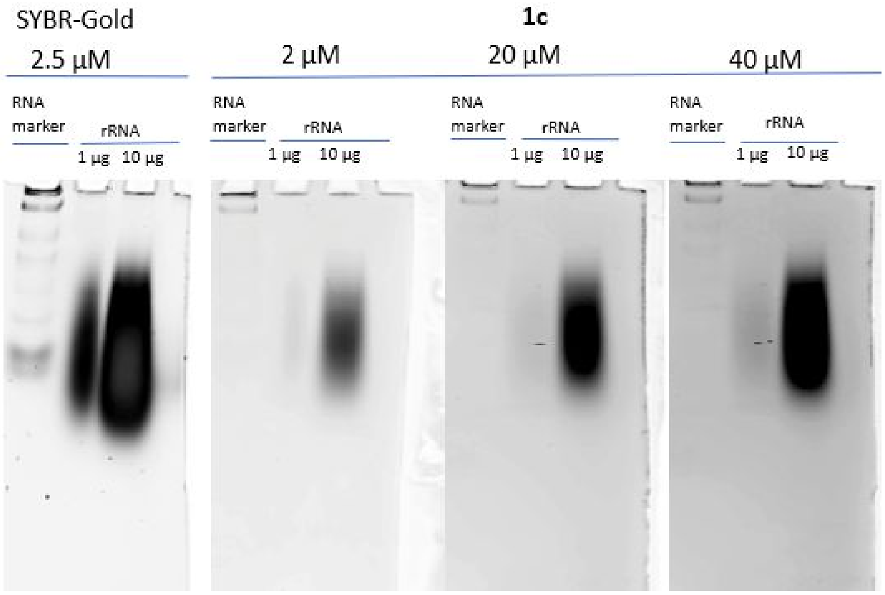

Figure S2. Staining of rRNA by **1c** or SYBR-Gold in PAGE-gels. Baker's yeast RNA was loaded and resolved in a native-PAGE gel before being stained by SYBR-Gold or **1c** at the indicated amounts.

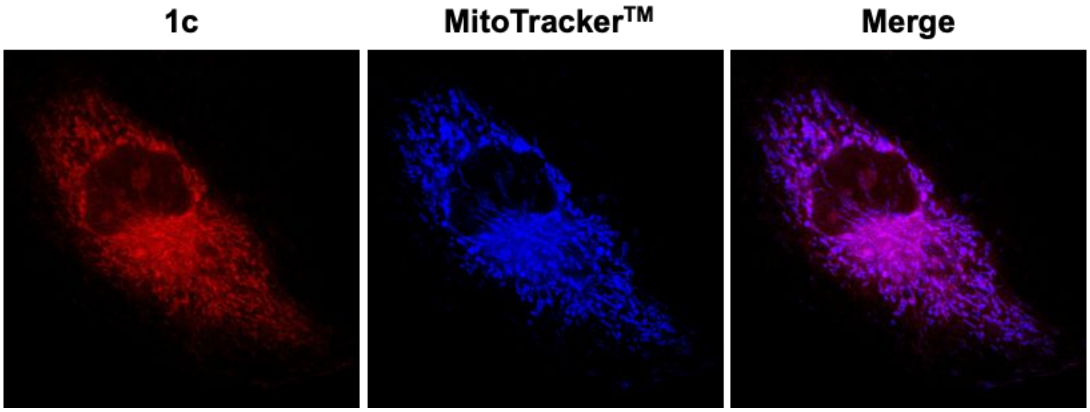

Figure S3. Zoomed in fluorescence image of live HeLa cells stained with 1 μM of **1c** and 0.5 μM MitoTracker™ Red CMXRos. The merged images indicate that **1c** and MitoTracker™ largely colocalize in the cytoplasm.

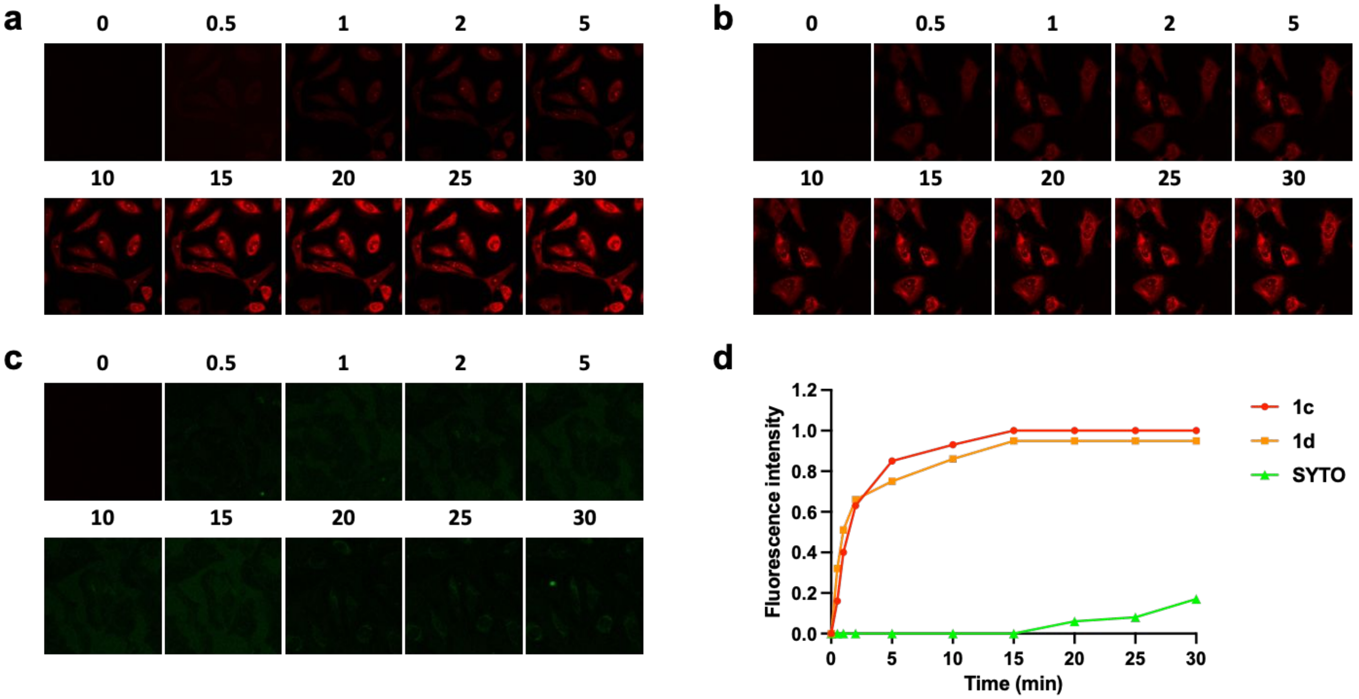

Figure S4. Time-resolved fluorescence images of live HeLa cell stained with 1 μM of (a) **1c**, (b) **1d**, and (c) SYTO. Images were taken at ten different time points within 30 mins. (d) Quantitative representation of the time-resolved imaging experiments.

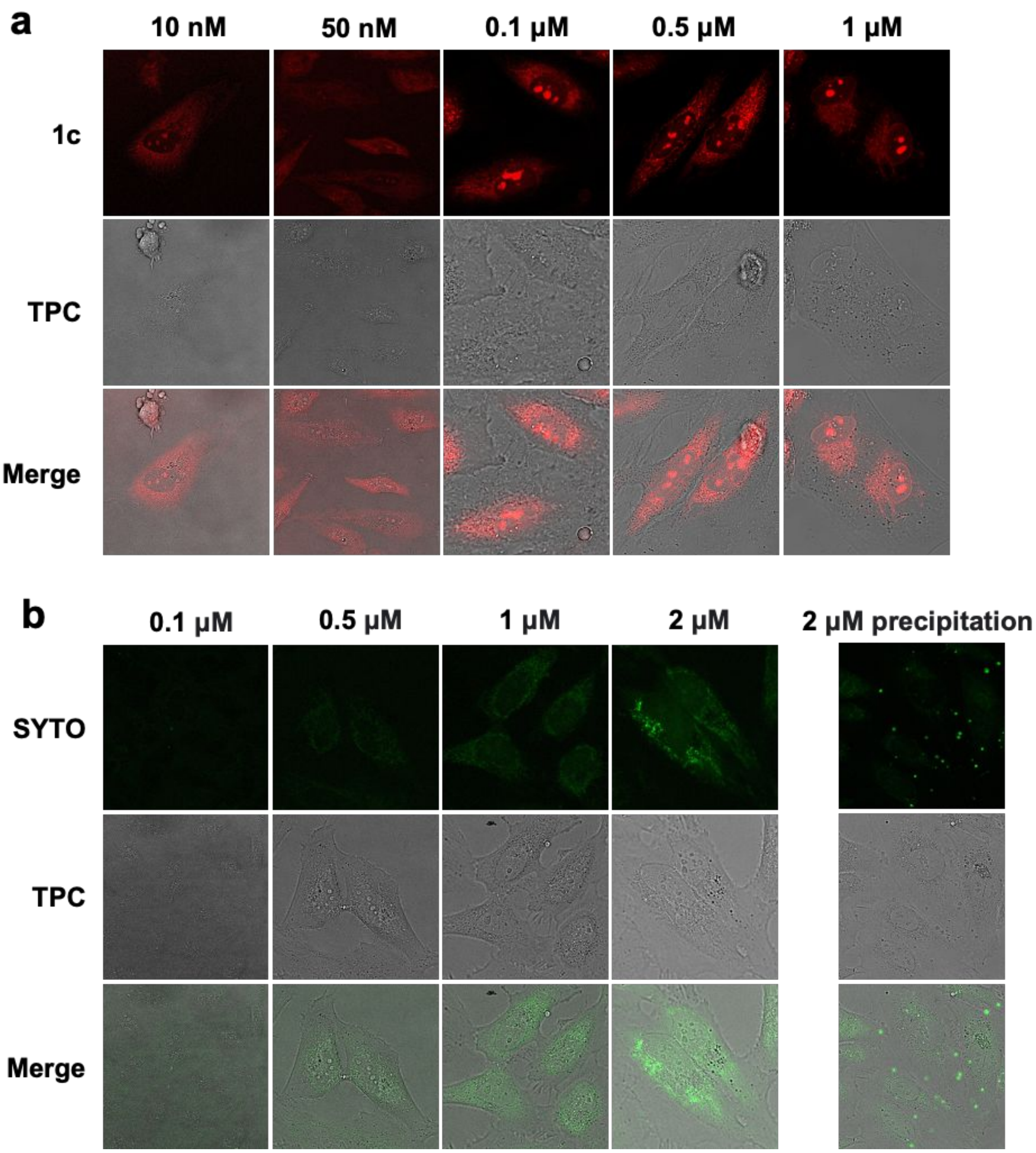

Figure S5. Fluorescence images of live HeLa cells stained with (a) 10 nM – 1 μM **1c** and (b) 0.1 – 2 μM of SYTO^{TM} RNASelect. At higher concentrations of SYTO (≥ 2 μM), dye aggregates were observed likely due to the largely hydrophobic nature of the dye.

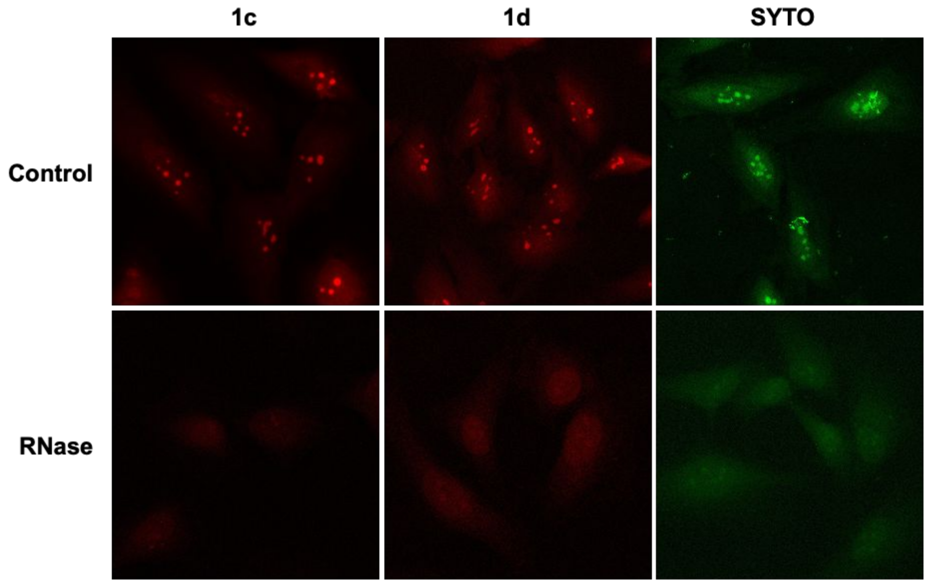

Figure S6. Fixed-permeabilized HeLa cells stained with 1 μM of **1c**, **1d**, or SYTO were treated with ribonuclease (RNase). The fluorescence intensity associated with each dye dramatically diminished upon RNase treatment.

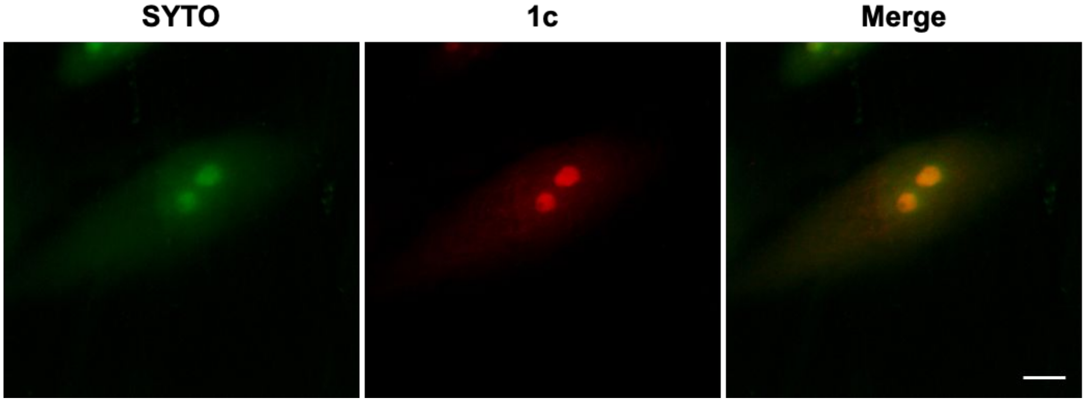

Figure S7. PFA-fixed HeLa cells were co-stained with SYTO (1 μM) and **1c** (0.2 μM). Regions of co-localization (yellow) can be seen in the merged image. Scale bar 10 μM.

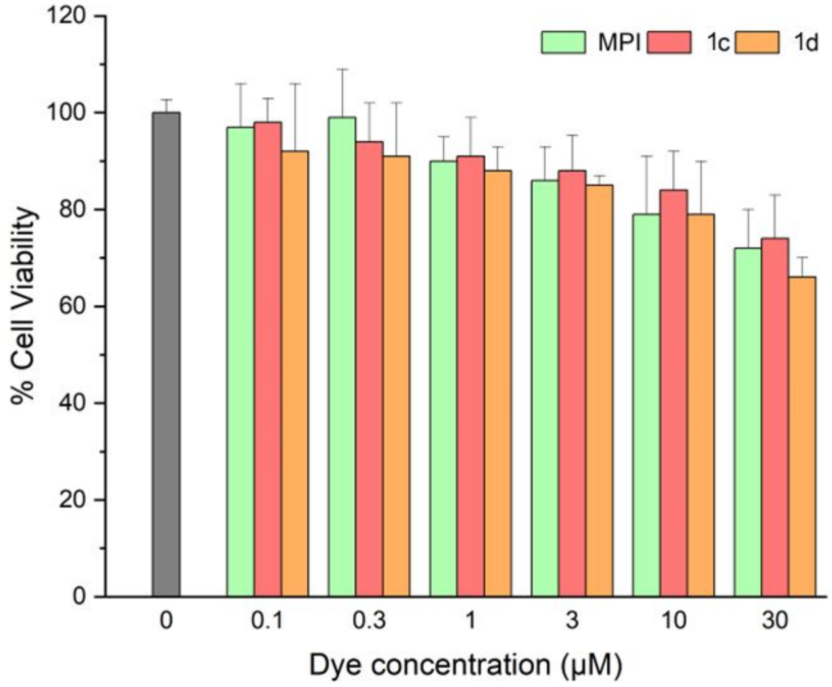

Figure S8. Cytotoxicity of MPI, **1c**, and **1d** in HeLa cells. Cells were treated with different concentrations of the dyes ranging from 0.1 to 30 μM for 24 hours before MTT dye was added to measure cell viability. Data are expressed as mean value ± standard deviation of three separate trials.

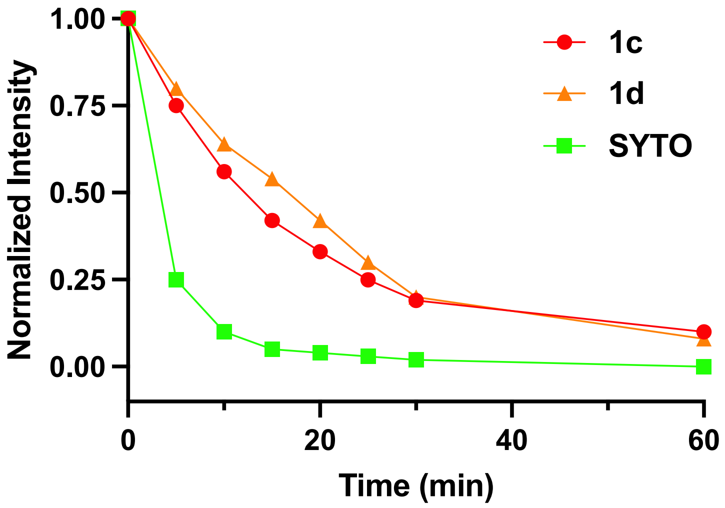

Figure S9. Quantitative representation of the photobleaching of SYTO^{TM} RNASelect, **1c**, and **1d** from photostability imaging experiments.

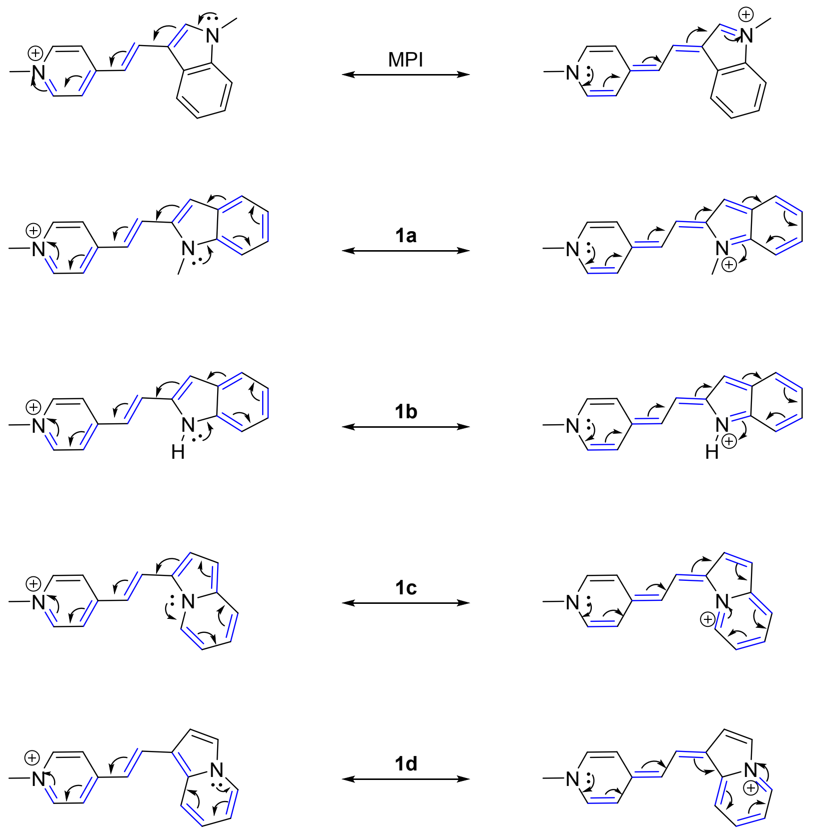

Figure S10. A graphical representation of the resonance structures of MPI, **1a**, **1b**, **1c**, and **1d**. Transitions between the resonance forms involve electron movement through π-conjugated systems of different lengths. Double bonds involved in the transition are colored blue.

Supplemental Videos

Supplemental Video 1. Dynamic imaging of live HeLa cells. The fiber-like structures in the cytoplasm represent mitochondria; these structures can be seen migrating, merging, and segmenting within the cytoplasm. Cells were stained with 1 μM of **1c.** Scale bar: 10 μm.

Supplemental Video 2. Dynamic imaging of live HeLa cells undergoing apoptosis. The captured video shows the formation of blebs, a hallmark of apoptotic cells, along the periphery of the cell membrane. Cells were stained with 1 μM of **1c**. Scale bar: 10 μm.

Materials & Methods

1. Chemical synthesis

1.1 Materials and instruments

1H-indole-2-carbaldehyde and 1-methyl-1H-indole-2-carbaldehyde were purchased from 1PlusChem and ChemScene. 2-pyridiniumpropanol was purchased from AAblocks. 4-methylpyridine was purchased from TCI. Iodomethane and 10% palladium on Charcoal were purchased from Sigma. All organic solvents used in the synthesis were HPLC grade and purchased from Sigma Millipore.

^{1}H NMR spectra were collected by using Varian Mercury-400 NMR spectrometer. ^{13}C NMR spectra were collected by using Varian VNMRS-600 NMR spectrometer. All NMR samples were dissolved in DMSO-d6. Mass spectrometry or LC−MS was performed on a Finnigan LCQ Deca XP Max equipped with an electrospray ionization (ESI) source (negative or positive ion mode) and a photodiode array (PDA) detector.

1.2 Synthetic procedures

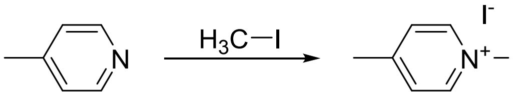

4-methylpyridinium iodide

4-methylpyridinium iodide. 4-Methylpyridine (10.2 mmol) was added to iodomethane (32 mmol) solution in Et_2O with stirring for 2 hours at room temperature. The product as solid was collected by vacuum filtration and used in subsequent reactions without further characterization[1].

(*E*)-4-(2-(1-Methyl-1H-indol-2-yl)vinyl)-1-methylpyridinium iodide (1a). A solution mixture of 4-methylpyridinium iodide (0.118 g, 0.5 mmol), 1-methyl-1H-indole-2-carbaldehyde (**2a**) (0.08 g, 0.5 mmol) and piperidine (0.07 mL) in MeOH (5 mL) was refluxed at 60 °C overnight. After cooling down to room temperature, the residue was filtered, washed with cold methanol to afford 0.15 g of a yellow product in 80% yield. ^{1}H NMR (400 MHz, DMSO-d6) δ 8.83 (d, *J* = 6.2 Hz, 2H), 8.28 (d, *J* = 6.3 Hz, 2H), 8.16 (d, *J* = 16.1 Hz, 1H), 7.62 (d, *J* = 7.9 Hz, 1H), 7.56 – 7.48 (m, 2H), 7.24 (d, *J* = 8.4 Hz, 2H), 7.09 (t, *J* = 7.5 Hz, 1H), 4.24 (s, 3H), 3.95 (s, 3H). ^{13}C NMR (500 MHz, DMSO-d6) δ 152.65, 145.24, 139.34, 136.75, 129.64, 127.63, 123.96, 123.68, 121.49, 120.81, 110.86, 103.59, 47.24 ppm. LC-MS (ESI) (m/z) calculated for $C_{17}H_{17}N_2^+$ $[M+H]^+$ 249.1, found 249.4.

(*E*)-4-(2-(1H-indol-2-yl)vinyl)-1-methylpyridinium iodide (1b). A solution mixture of 4-methylpyridinium iodide (0.074 g, 0.31 mmol), 1H-indole-2-carbaldehyde (**2b**) (0.05 g, 0.31 mmol) and piperidine (0.045 mL) in MeOH (3.2 mL) was refluxed at 60 °C overnight. After cooling down to room temperature, the residue was filtered, washed with cold methanol to afford 0.093 g of a yellow product

in 81% yield. ^{1}H NMR (400 MHz, DMSO-d6) δ 11.72 (s, 1H), 8.77 (d, *J* = 6.3 Hz, 2H), 8.12 (d, *J* = 6.3 Hz, 2H), 7.99 (d, *J* = 16.2 Hz, 1H), 7.60 (d, *J* = 8.0 Hz, 1H), 7.41 (d, *J* = 8.3 Hz, 1H), 7.27 (d, *J* = 16.1 Hz, 1H), 7.21 (t, *J* = 7.7 Hz, 1H), 7.03 (t, *J* = 7.6 Hz, 1H), 6.90 (s, 1H), 4.22 (s, 3H). ^{13}C NMR (500 MHz, DMSO-d6) δ 152.66, 145.31, 138.95, 135.43, 131.77, 128.46, 124.79, 123.39, 121.71, 121.33, 120.44, 112.01, 109.41, 47.22 ppm. LC-MS (ESI) (m/z) calculated for $C_{16}H_{15}N_2^+$ $[M+H]^+$ 235.1, found 235.3.

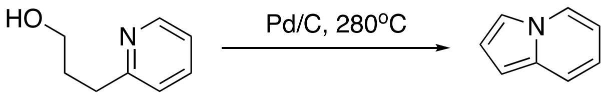

Indolizine. A mixture of 2-pyridinepropanol (1 mL, 7.27 mmol) and palladium on activated carbon (10%) (15 mg, 10 mmol%) was refluxed under N_2 atmosphere at 280 °C for 8 hours. The residue was filtered and washed with MeOH. The filtrate was collected and concentrated *in vacuo*. The solution was purified by Flash chromatography to afford 111 mg of a white solid in 13% yield. The product was used in subsequent reactions without further characterization[2].

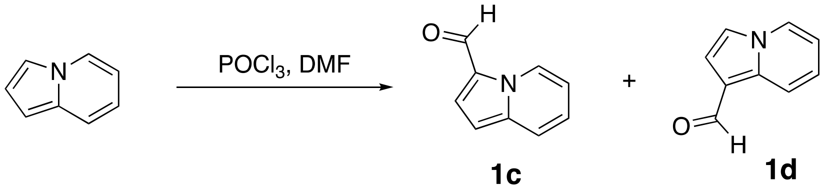

1c 1d

3-indolizinecarboxaldehyde (2c) and **1-indolizinecarboxaldehyde (2d).** To a stirred solution of indolizine (**1**) (0.1 g, 0.93 mmol) in 1.2 mL DMF was added dropwise a 3 M $POCl_3$ solution in DMF. The mixture was stirred for 2 hours at room temperature. After stirring, 1.5 mL of ice water and 1.1 mL of 25% NaOH was added. The mixture was refluxed for 10 minutes. The filtrate was extracted with chloroform and dried over Na_2CO_3 and concentrated *in vacuo*. The concentrated mixture was purified by flash chromatography to afford 63 mg of compound **2c** as dark-blue oil in 47% yield and 15 mg of compound **2d** as dark-blue oil in 12% yield[3].

(*E*)-4-(2-(indolizin-3-yl)vinyl)-1-methylpyridinium iodide (1c). A solution mixture of 4-methylpyridinium iodide (0.1 g, 0.43mmol), 3-indolizinecarbaldehyde (**2c**) (0.063 g, 0.39 mmol) and piperidine (0.06 mL) in MeOH (4 mL) was refluxed at 60 °C overnight. After cooling down to room temperature, the residue was filtered, washed with cold methanol to afford 0.083 g of a dark blue product in 59% yield. ^{1}H NMR (400 MHz, DMSO-d6) δ 8.95 (d, *J* = 7.1 Hz, 1H), 8.61 (d, *J* = 6.7 Hz, 2H), 8.46 (d, *J* = 15.5 Hz, 1H), 8.05 (d, *J* = 6.7 Hz, 2H), 7.60 (d, *J* = 8.8 Hz, 1H), 7.55 (d, *J* = 4.5 Hz, 1H), 7.14 (d, *J* = 15.5 Hz, 1H), 7.09 – 7.00 (m, 1H), 6.93 (t, *J* = 6.8 Hz, 1H), 6.72 (d, *J* = 4.5 Hz, 1H),

4.12 (s, 3H). ^{13}C NMR (500 MHz, DMSO-d6) δ 153.78, 144.34, 137.73, 127.77, 125.19, 123.76, 122.13, 121.93, 119.99, 117.43, 115.91, 113.48, 105.16, 46.55 ppm. LC-MS (ESI) (m/z) calculated for $C_{16}H_{15}N_2^+$ $[M+H]^+$ 235.1, found 235.4.

(*E*)-4-(2-(indolizin-1-yl)vinyl)-1-methylpyridinium iodide (1d). A solution mixture of 4-methylpyridinium iodide (0.025g, 0.11 mmol), 1-indolizinecarbaldehyde (**2d**) (0.015 g, 0.1 mmol) and piperidine (0.02 mL) in MeOH (1 mL) was refluxed at 60 °C overnight. After cooling down to room temperature, the residue was filtered, washed with cold methanol to afford 0.014 g of a dark blue product in 40% yield. ^{1}H NMR (400 MHz, DMSO-d6) δ 8.57 (d, *J* = 6.6 Hz, 2H), 8.41 (dd, *J* = 6.9, 1.3 Hz, 1H), 8.37 (d, *J* = 15.7 Hz, 1H), 8.09 (d, *J* = 9.0 Hz, 1H), 8.00 (d, *J* = 6.7 Hz, 2H), 7.72 (d, *J* = 3.1 Hz, 1H), 7.28 (d, *J* = 3.1 Hz, 1H), 7.12 (ddd, *J* = 9.1, 6.6, 1.1 Hz, 1H), 7.00 (d, *J* = 15.7 Hz, 1H), 6.81 (td, *J* = 6.8, 1.2 Hz, 1H), 4.11 (s, 3H).^{13}C NMR (500 MHz, DMSO-d6) δ 154.26, 144.23, 135.30, 134.58, 128.11, 122.92, 121.55, 118.12, 117.83, 115.64, 113.23, 112.51, 111.71, 46.42 ppm. LC-MS (ESI) (m/z) calculated for $C_{16}H_{15}N_2^+$ $[M+H]^+$ 235.1, found 235.4.

1.3 NMR spectra

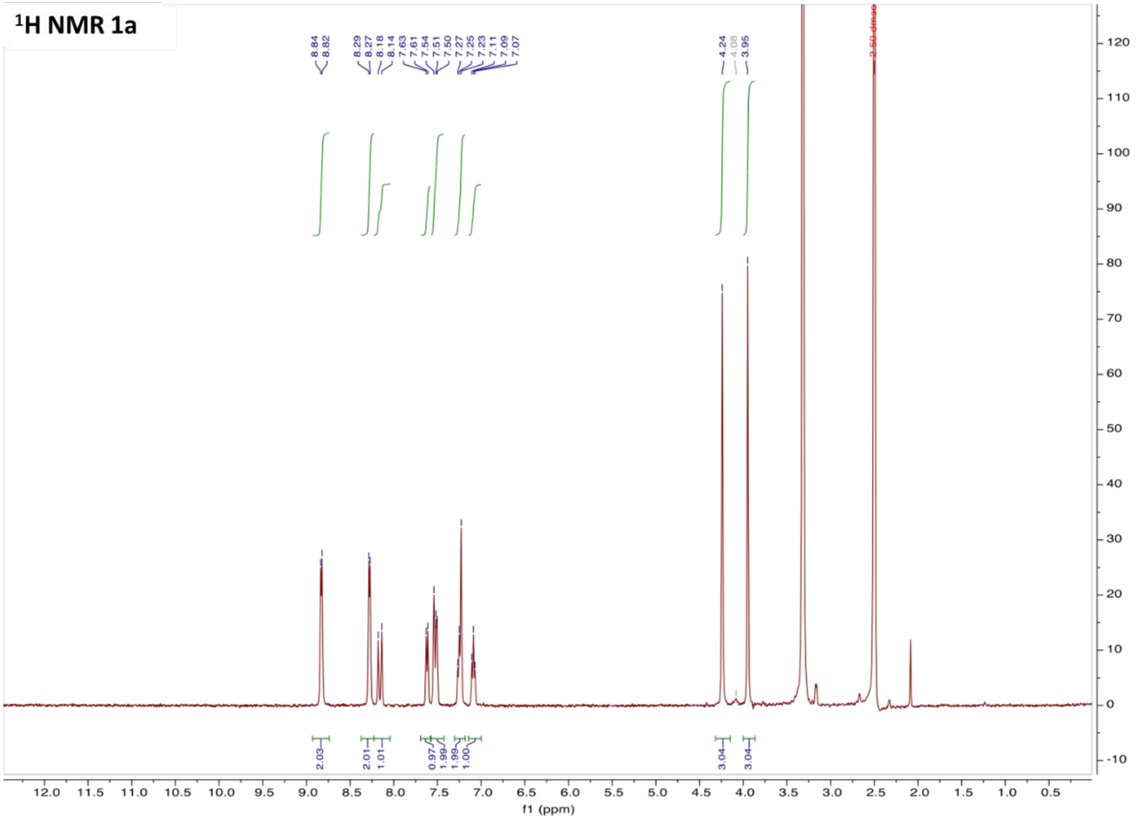

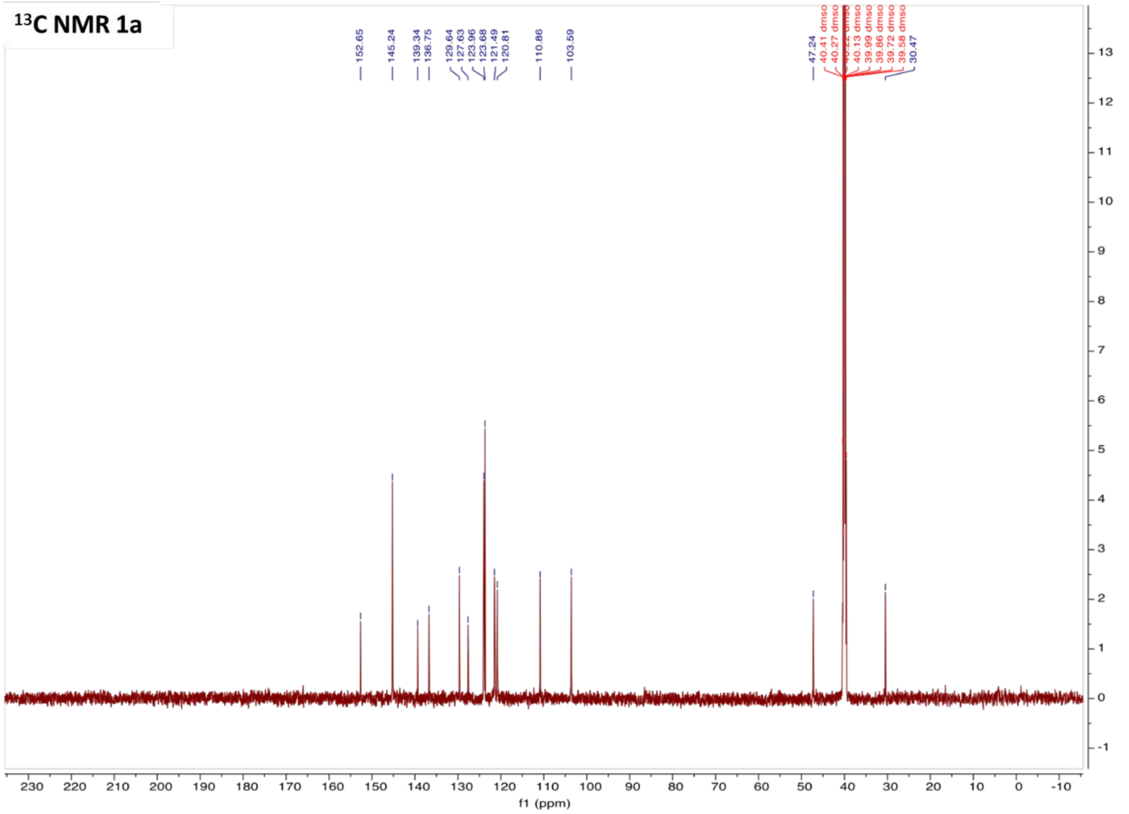

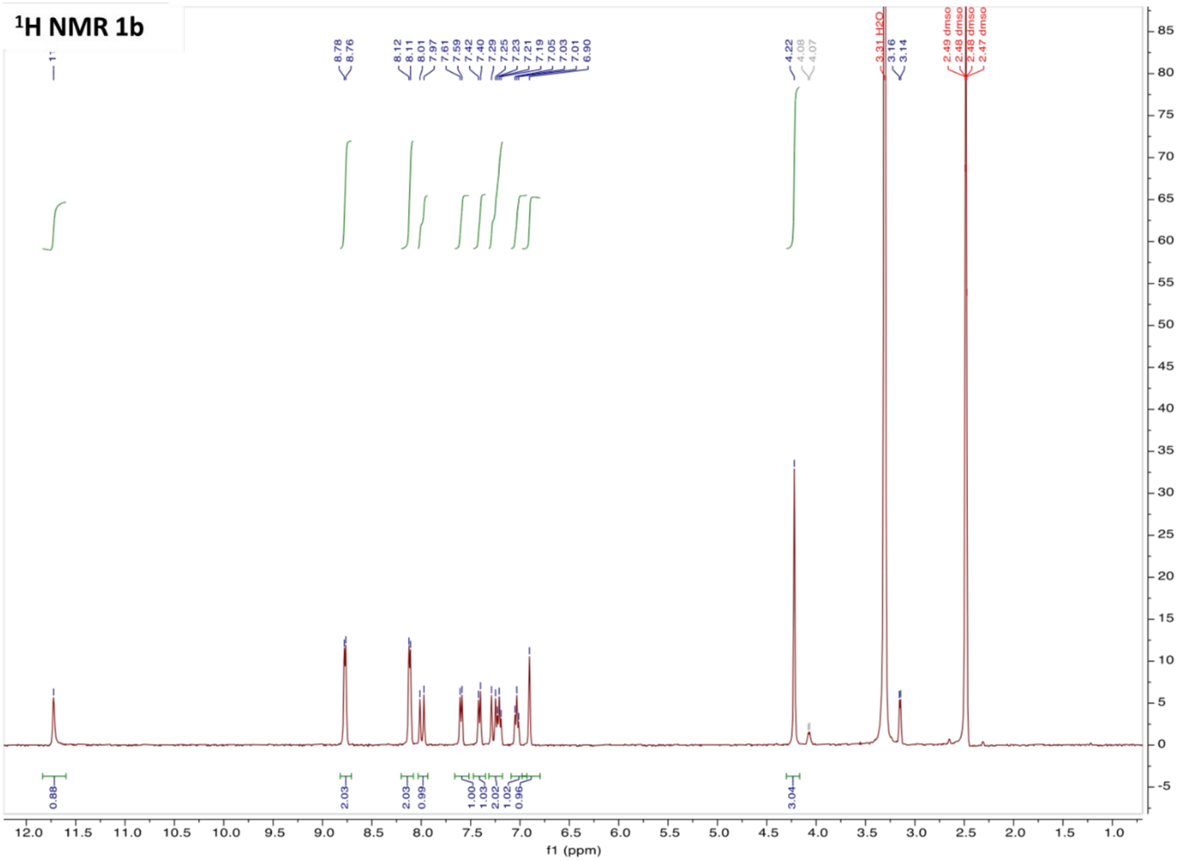

1H NMR 1b
f1 (ppm)

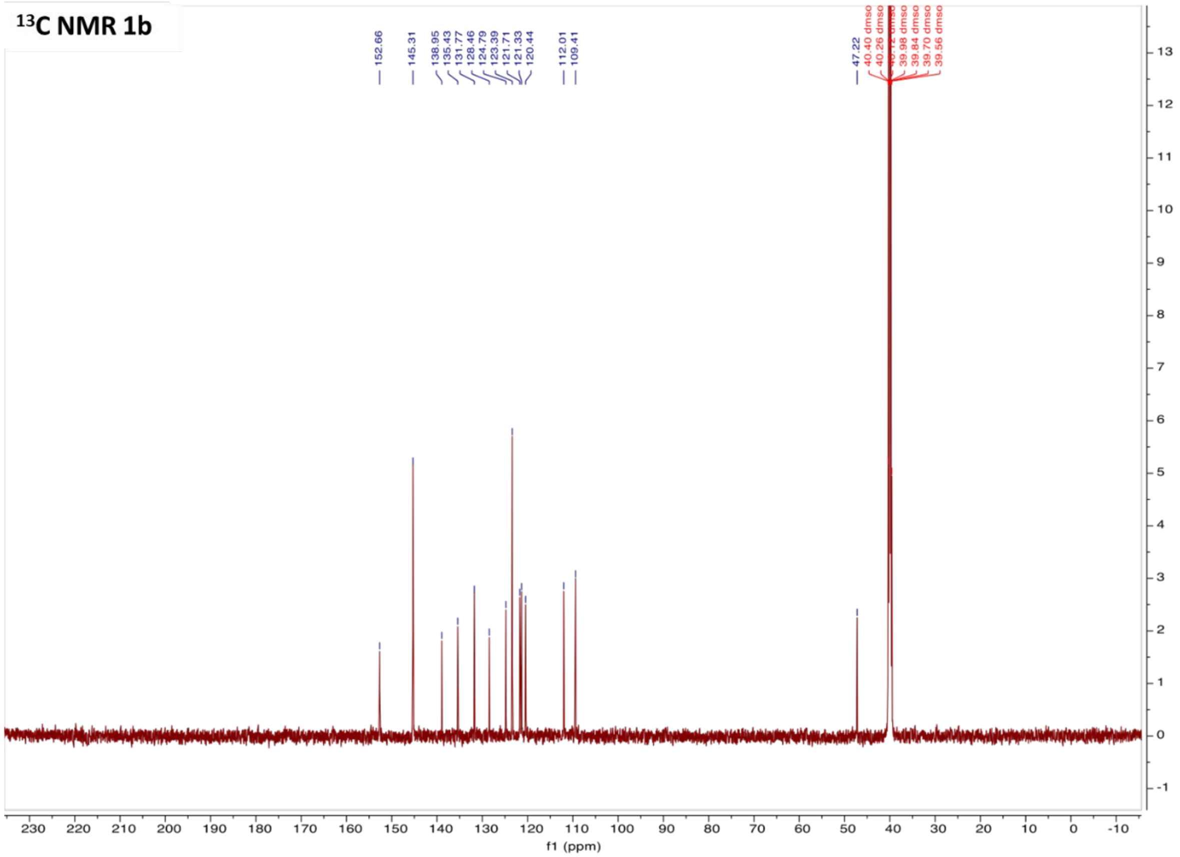

13C NMR 1b
f1 (ppm)

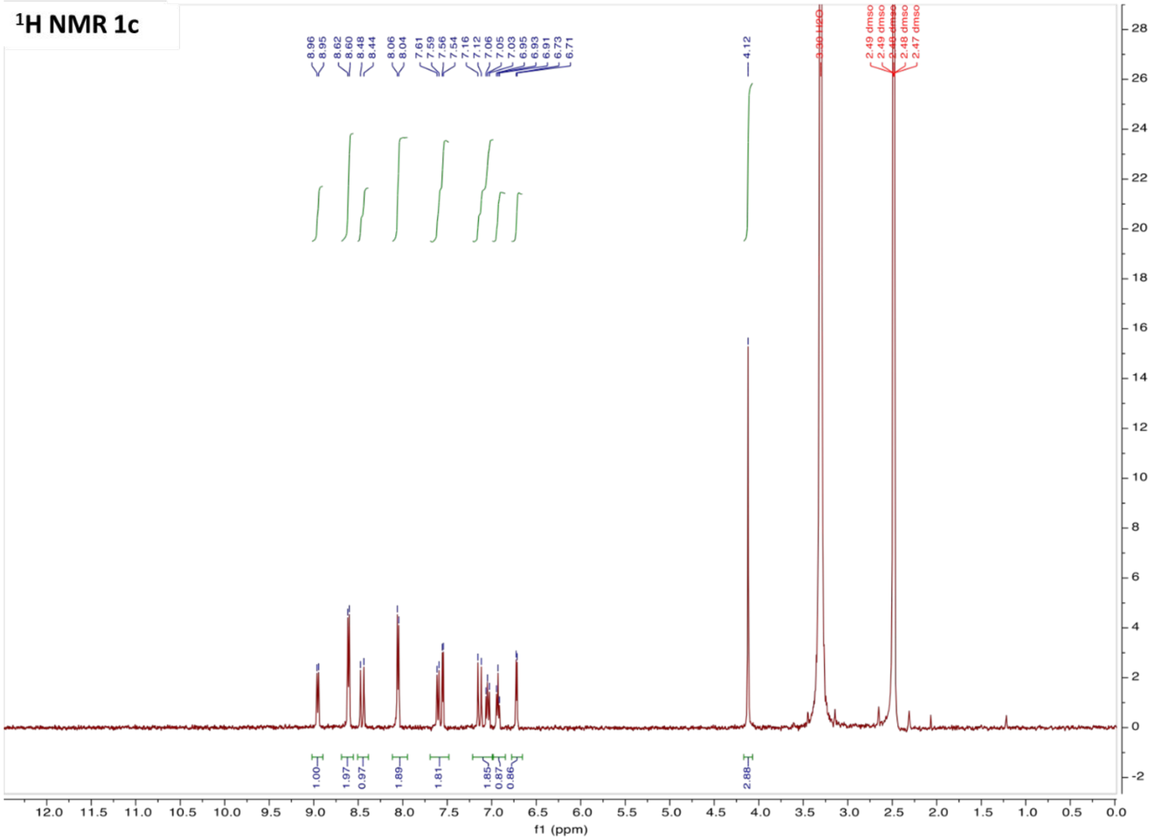
1H NMR 1c
8.96
8.95
8.62
8.60
8.48
8.44
8.06
8.04
7.61
7.59
7.56
7.54
7.16
7.12
7.06
7.05
7.03
6.95
6.93
6.91
6.73
6.71
4.12
3.30 H2O
2.49 dmso
2.49 dmso
2.48 dmso
2.48 dmso
2.47 dmso
1.00
1.97
0.97
1.89
1.81
1.85
0.87
0.86
2.88
f1 (ppm)

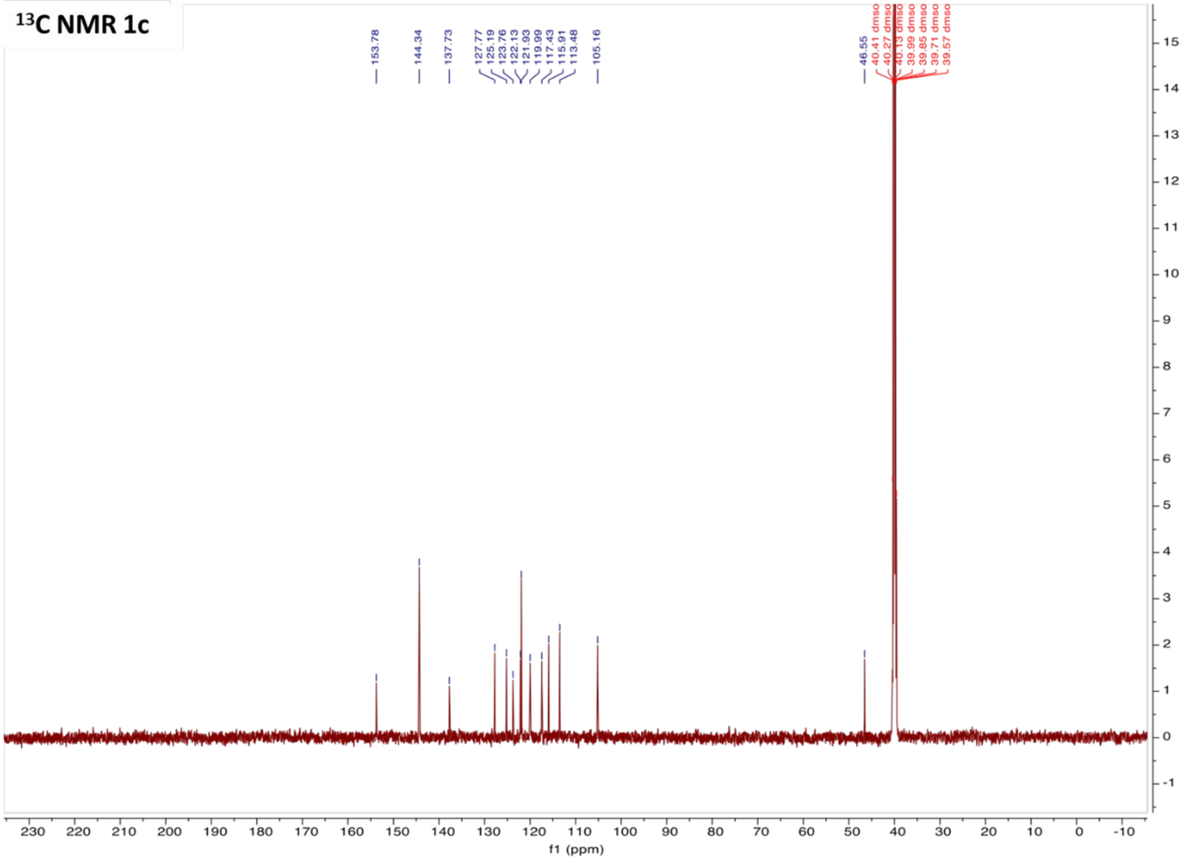
13C NMR 1c
153.78
144.34
137.73
127.77
125.19
123.76
122.13
121.93
119.99
117.43
115.91
113.48
105.16
46.55
40.41 dmso
40.27 dmso
40.13 dmso
39.99 dmso
39.85 dmso
39.71 dmso
39.57 dmso
f1 (ppm)

^{1}H NMR 1d

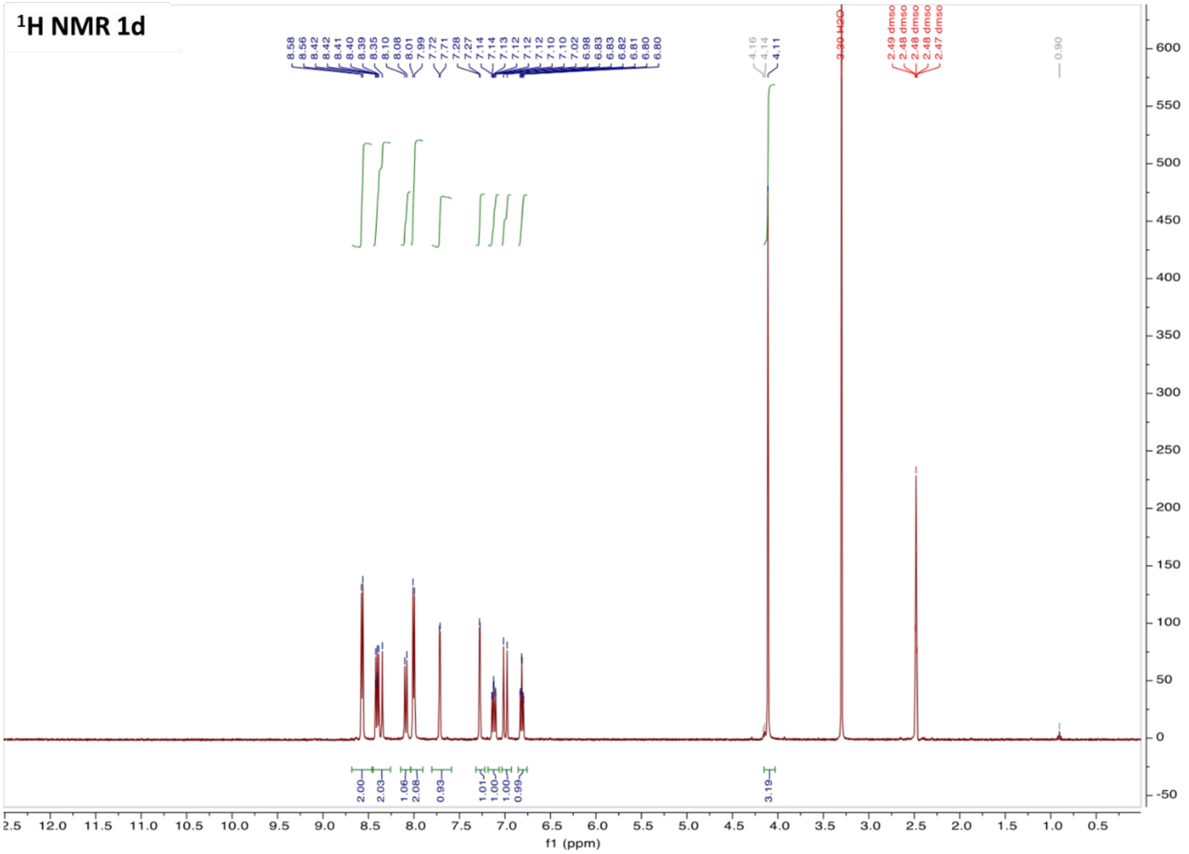

^{13}C NMR 1d

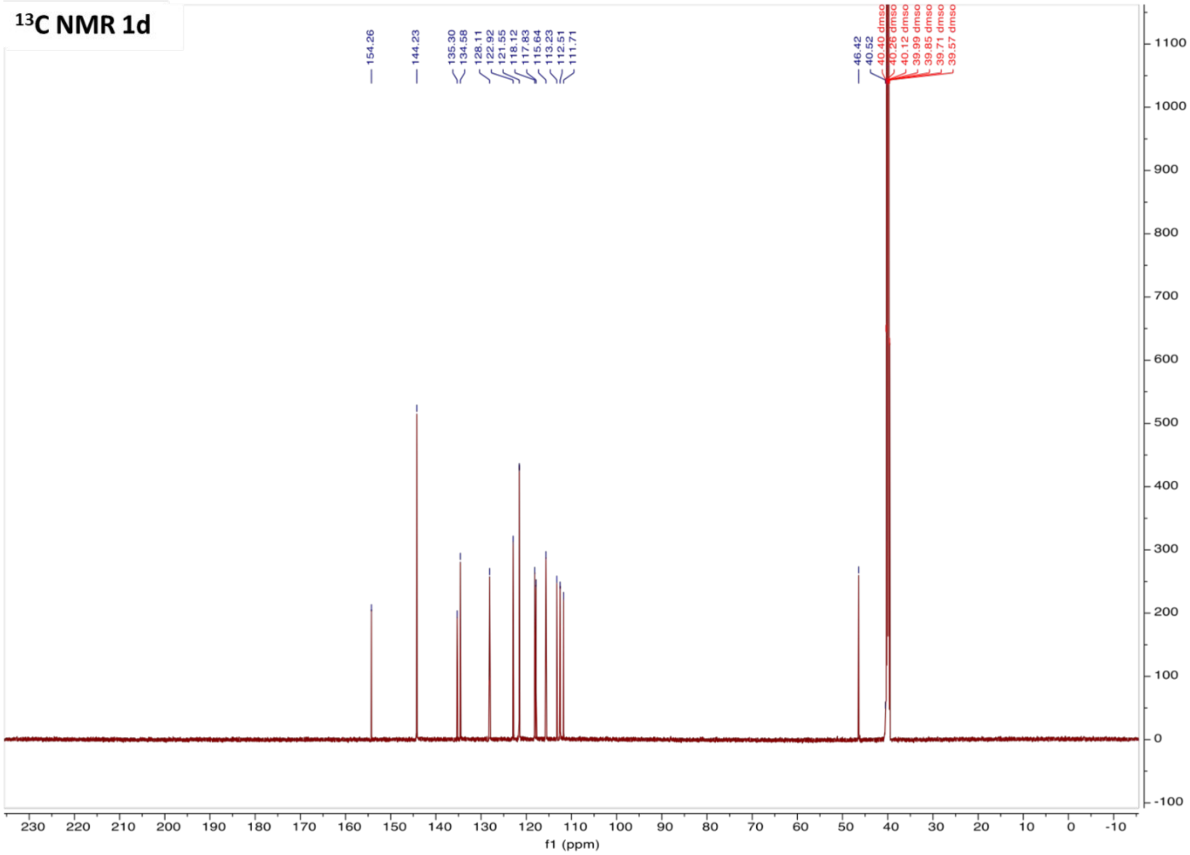

1.4 MS spectra

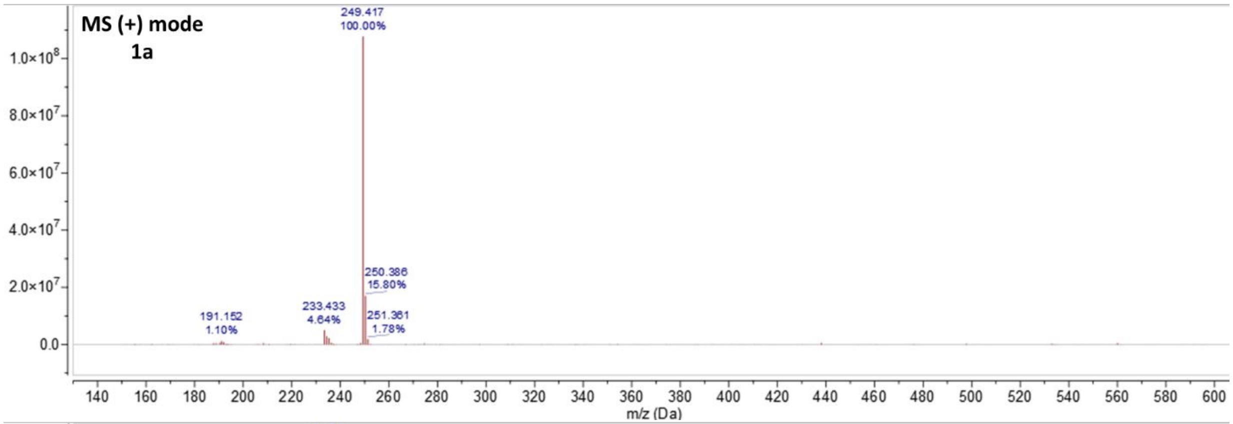

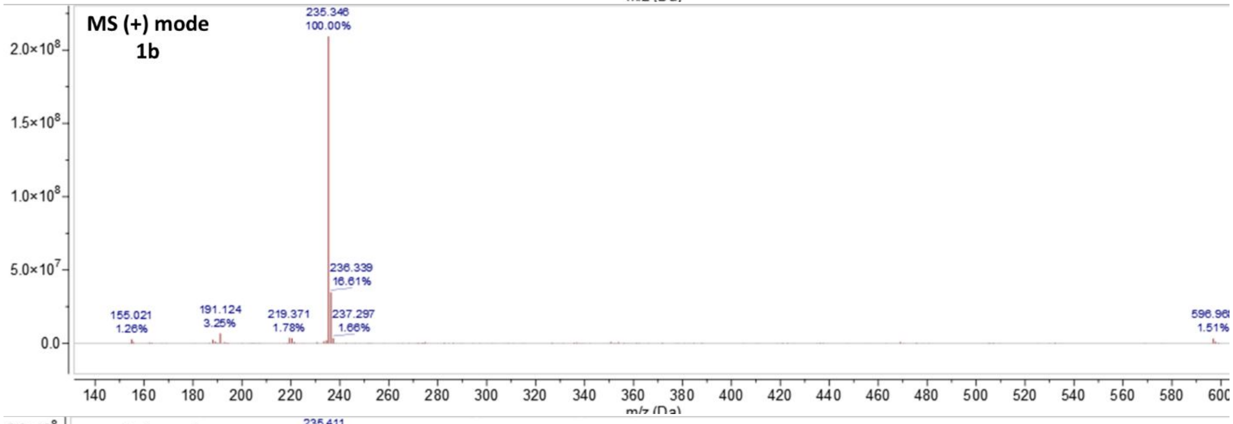

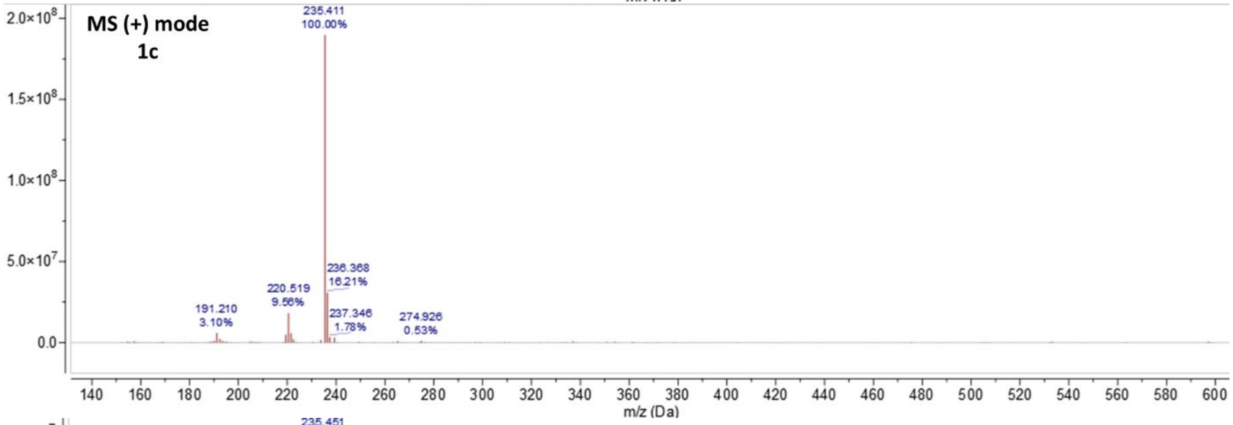

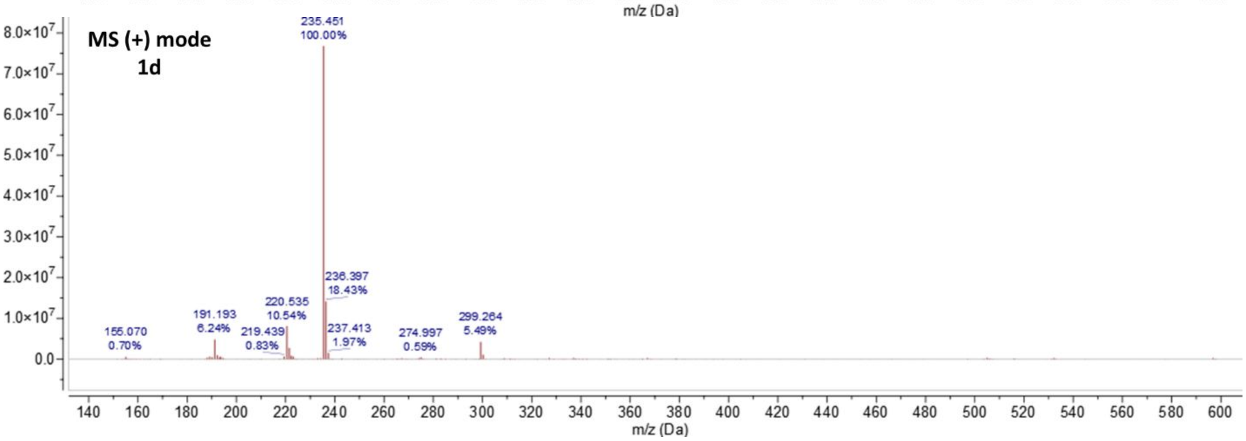

References

1. Ustimova, M. A.; Chernikova, P. A.; Shepel, N. E.; Fedorov, Y. V.; Fedorova, O. A. Effect of N-Substituent in 4-Styrylpyridinium Dyes on Their Binding to DNA. *Mendeleev Communications* **2020**, *30* (2), 217–219.
2. Boekelheide, V.; Windgassen, R. J. Syntheses of Pyrrocolines Unsubstituted in the Five-Membered Ring1. *Journal of the American Chemical Society* **1959**, *81* (6), 1456–1459.
3. Fuentes, O.; Paudler, W. W. Some Formylation Reactions of Imidazo[1,5-*a*] Pyridine and Pyrrocoline. *Journal of Heterocyclic Chemistry* **1975**, *12* (2), 379–383.
4. Bohler, P. et al. The mycotoxin phomoxanthone A disturbs the form and function of the inner mitochondrial membrane. *Cell Death Dis* **2018,** *9* (3), 286.
